# Novel molecular mechanism driving neuroprotection after soluble epoxide hydrolase inhibition: Insights for Alzheimer's disease therapeutics

**DOI:** 10.1111/cns.14511

**Published:** 2023-10-31

**Authors:** Christian Griñán‐Ferré, Júlia Jarné‐Ferrer, Aina Bellver‐Sanchís, Sandra Codony, Dolors Puigoriol‐Illamola, Coral Sanfeliu, Yumin Oh, Seulki Lee, Santiago Vázquez, Mercè Pallàs

**Affiliations:** ^1^ Department of Pharmacology and Therapeutic Chemistry Institut de Neurociències‐Universitat de Barcelona Barcelona Spain; ^2^ Centro de Investigación en Red, Enfermedades Neurodegenerativas (CIBERNED), Instituto de Salud Carlos III Madrid Spain; ^3^ Laboratory of Medicinal Chemistry (CSIC Associated Unit), Faculty of Pharmacy and Food Sciences, Institute of Biomedicine (IBUB) University of Barcelona (UB) Barcelona Spain; ^4^ Institut d'Investigacions Biomèdiques de Barcelona (IIBB), Consejo Superior de Investigaciones Científicas (CSIC) Barcelona Spain; ^5^ Neuraly Inc. Maryland Gaithersburg USA

**Keywords:** 5XFAD, endoplasmic reticulum stress, epoxide hydrolase, glia, neuroinflammation

## Abstract

**Background:**

Neuroinflammation is widely recognized as a significant hallmark of Alzheimer's disease (AD). To combat neuroinflammation, the inhibition of the soluble epoxide hydrolase (sEH) enzyme has been demonstrated crucial. Importantly, sEH inhibition could be related to other neuroprotective pathways described in AD.

**Aims:**

The aim of the study was to unveil new molecular pathways driving neuroprotection through sEH, we used an optimized, potent, and selective sEH inhibitor (sEHi, UB‐SCG‐51).

**Materials and Methods:**

UB‐SCG‐51 was tested in neuroblastoma cell line, SH‐SY5Y, in primary mouse and human astrocytes cultures challenged with proinflammatory insults and in microglia cultures treated with amyloid oligomers, as well as in mice AD model (5XFAD).

**Results:**

UB‐SCG‐51 (10 and 30 μM) prevented neurotoxic reactive‐astrocyte conversion in primary mouse astrocytes challenged with TNF‐α, IL‐1α, and C1q (T/I/C) combination for 24 h. Moreover, in microglial cultures, sEHi reduced inflammation and glial activity. In addition, UB‐SCG‐51 rescued 5XFAD cognitive impairment, reducing the number of Amyloid‐β plaques and Tau hyperphosphorylation accompanied by a reduction in neuroinflammation and apoptotic markers. Notably, a transcriptional profile analysis revealed a new pathway modulated by sEHi treatment. Specifically, the eIF2α/CHOP pathway, which promoted the endoplasmic reticulum response, was increased in the 5XFAD‐treated group. These findings were confirmed in human primary astrocytes by combining sEHi and eIF2α inhibitor (eIF2αi) treatment. Besides, combining both treatments resulted in increased in C3 gene expression after T/I/C compared with the group treated with sEHi alone in cultures.

**Discussion:**

Therefore, sEHi rescued cognitive impairment and neurodegeneration in AD mice model, based on the reduction of inflammation and eIF2α/CHOP signaling pathway.

**Conclusions:**

In whole, our results support the concept that targeting neuroinflammation through sEH inhibition is a promising therapeutic strategy to fight against Alzheimer's disease with additive and/or synergistic activities targeting neuroinflammation and cell stress.

## Graphical abstract

sEH inhibition reduced pro‐inflammatory processes in vitro and in vivo models, being the eIF2α/CHOP, an undescribed implicated neuroprotective pathway improving cognition and AD hallmarks in 5XFAD mice model by favoring synaptic plasticity, modulating ISR, and apoptosis.

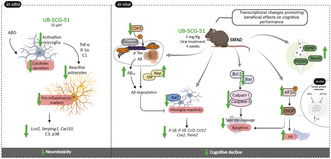

## 1 INTRODUCTION

Alzheimer's disease (AD) is the most prevalent form of dementia globally, with cognitive decline as the main symptom.^1^ AD is characterized by the presence of extracellular accumulation of the amyloid‐β (Aβ) plaques and the abnormal Tau phosphorylation (*p*‐Tau), forming neurofibrillary tangles (NFTs), in the brain.^2^, ^3^ However, both neuropathological AD hallmarks cannot explain all the pathogenesis of AD, suggesting the involvement of other pathological events. Those AD hallmarks initiate a range of brain molecular alterations mediated by the activation of neuroinflammation, apoptosis, and neuronal death, among others.^4^ Altogether, these alterations cause a progressive neurodegeneration process.^5^ However, despite the extensive research in this field, many clinical trials have been centered exclusively on the Aβ hypothesis, and at present, the molecular mechanisms triggering neurodegeneration are largely unclear. Thus, there is an urgent need to understand the etiopathogenesis of AD to develop novel disease‐modifying therapies.^6^

Growing evidence shows that uncontrolled neuroinflammation is well identified in many pathological conditions, which would impair the typical structures and functionality of neurons.^7^ Importantly, neuroinflammation is mediated by reactive glial cells (microglia and astrocytes), which are mainly activated by Aβ plaques.^8^, ^9^ Those cells promote neuroinflammation, controlling physiological response, by producing different inflammatory mediators that alter the microenvironment.^10^ Most notably, prolonged glial activation causes neurotoxicity and initiates a neurodegenerative cascade, which contributes to cognitive and synaptic impairment as well as endoplasmic reticulum stress (ERS).^11^ In addition, the complement system, a major effector mechanism of the immune system, can be activated, promoting microglial phagocytosis, and resulting in synaptic dysfunction.^12^ Therefore, the suppression of glial cell‐mediated inflammation has been proposed as an important strategy in neurodegenerative disease.

Epoxyeicosatrienoic acids (EETs) and epoxy‐fatty acids (EpFAs) are derivatives of arachidonic acid endowed with potent anti‐inflammatory properties.^13^, ^14^, ^15^, ^16^, ^17^ Interestingly, increased levels of EETs have been associated with microglial attenuation in AD mice models.^18^, ^19^ Thus, EETs are potential neuroprotective agents, which are known to modulate several neurodegeneration‐associated molecular pathways such as inflammatory, apoptotic, angiogenic, and oxidative stress, among others.^17^, ^20^ However, there is an ubiquitous enzyme in vertebrates (EC 3.3.2.10, *EPHX2*),^21^, ^22^ called soluble epoxide hydrolase (sEH), which promotes the principal route of EETs degradation into their corresponding less‐active diol metabolites, the dihydroeicosatrienoic acids (DHETs).^21^, ^22^ Finally, it has been demonstrated that sEH expression is increased in AD patients' brain and AD mice models, altering the anti‐inflammatory effects of EETs and boosting the DHETs.^22^

Given the above exposed, we and others^23^, ^24^ have recently proposed the sEH as a new target for a novel approach to AD treatment. Genetic ablation of the sEH gene in mice models delayed the progression of AD by alleviating behavior outcomes and AD pathological hallmarks.^25^ Moreover, it has been demonstrated that treatment with a sEH inhibitor (sEHi) in mice blocks the EpFA degradation and stabilizes EETs and other oxylipins levels in vivo.^26^ Furthermore, sEHi reduced cognitive impairment, Aβ plaques, *p*‐Tau, and neuroinflammation markers in AD mice models.^23^, ^24^, ^25^ Altogether, anticipate sEH inhibition as a successful approach to AD and other neurodegenerative diseases, serving as a potential therapeutic target.^23^ However, the neuroprotection described could benefit from other neuroprotection mechanisms that would explain the beneficial effects observed in different animal models. To move forward in this regard, the main goal of this work is to unveil further the mechanisms that mediate the neuroprotective role for sEH inhibition by using UB‐SCG‐51 (called Cpd 22 in the referenced article), an optimized sEHi.^27^ Additionally, we emphasized the effectiveness of targeting a network of pathways instead of a single pathway, such as the Aβ pathway, to fight against progressive neurodegeneration and cognitive impairment in AD. Lastly, we performed a transcriptome analysis using RNA‐sequencing (RNA‐seq), to clarify the precise mechanisms underlying neuroprotection after sEH inhibition. Importantly, the mechanisms modulated by the sEHi were focused on classical neurodegenerative pathways, especially neuroinflammation, apoptosis, ERS, and synaptic plasticity, which explain the positive outcomes found in vitro and in vivo AD models.

## 2 MATERIALS AND METHODS

### 2.1 Cells

#### 2.1.1 SH‐SY5Y cells

The human neuroblastoma cell line, SH‐SY5Y, was obtained from the American Type Culture Collection (ATCC). SH‐SY5Y cells were cultured on 96‐well plates (5 × 10^4^), in Dulbecco's modified Eagle's medium (DMEM) (Gibco) supplemented with nonessential amino acids, 10% fetal bovine serum (Gibco), and 1% penicillin–streptomycin (Gibco) at 37°C, in a 5% CO_2_ atmosphere.

#### 2.1.2 Microglial cultures

A total quantity of 3 × 10^5^ microglia cells isolated from CD1 mouse brain (ScienCell #M1900) were seeded onto poli‐l‐lysine‐coated 12‐well culture plates in microglia medium (ScienCell #1901).

#### 2.1.3 Astrocyte cultures

A total quantity of 1 × 10^6^ astrocytes isolated from CD1 mouse brain (ScienCell #M1800) or primary human astrocyte (ScienCell #1800) were seeded onto PLL‐coated 6‐well culture plates in astrocyte medium (ScienCell #1831 or #1801).

### 2.2 Animals

5XFAD (*n* = 48) and wild‐type (WT, *n* = 16) female mice (7‐month‐old) were used. Animals were randomly divided into WT Control (WT Ct) (*n* = 16), 5XFAD Control (5XFAD Ct) (*n* = 16), and 5XFAD treated with UB‐SCG‐51 at 5 mg/Kg dose^23^ (5XFAD UB‐SCG‐51) (*n* = 16). UB‐SCG‐51 was administered through drinking water, and dosages were calculated based on average daily water consumption recorded in each cage weekly. Control groups received water plus 1.8% (2‐hydroxypropyl)‐β‐cyclodextrin (vehicle) during the treatment period. The animals had free access to food and water and were kept under standard temperature conditions (22 ± 2°C) and 12‐h/12‐h light/dark cycles (300 lux/0 lux).

After 4 weeks of treatment, behavioral and cognitive tests were performed. During this period and up to the killing, mice received UB‐SCG‐51 or vehicle. All studies and procedures for the mouse behavior tests, brain dissection, and extractions followed the ARRIVE standard ethical guidelines (European Communities Council Directive 2010/63/EU and Guidelines for the Care and Use of Mammals in Neuroscience and Behavioral Research, National Research Council 2003) and were approved by the Institutional Animal Care and Generalitat de Catalunya (#10291, 1/28/2018). All efforts were made to minimize the number of mice used and their suffering.

### 2.3 In vitro experiments

#### 2.3.1 Cell viability assay

SH‐SY5Y cells were equilibrated to room temperature for 30 min and followed by UB‐SCG‐51 treatment (100 μM) or PBS for 24 h. A total quantity of 50 μL of Cell titer Glo (Madison) reagent was added to each well and incubated for 10 min. The luminescence of each sample was measured on a plate reader (BioTek, Agilent) with parameters of 1 min lag time and 0.5 s/well‐read time (*n* = 3).

#### 2.3.2 Treatment of primary microglial cultures with Aβ oligomers (AβO)

Microglia cultures were incubated in serum‐free condition for 24 h and pretreated for 30 min with UB‐SCG‐51 (3 or 10 μM). Afterward, AβO [1 μM, Aβ (1–42), Ultra‐Pure, HFIP A‐1163‐1, rPeptide] or phosphate saline buffer were added for 4 h.

#### 2.3.3 Treatment of astrocyte with T/I/C

Astrocytes cultures were incubated in serum‐free condition for 24 h and pretreated for 30 min with UB‐SCG‐51 or *p*‐eIF2α signal inhibitor (Sigma, SML0843), followed by recombinant T/I/C: IL‐1α (3 ng/mL, Peprotech, Thermo Fisher), TNF‐α (30 ng/mL, R&D), C1q (400 ng/mL, R&D), or PBS for 24 h.

### 2.4 In vivo experiments

UB‐SCG‐51 was dissolved in 2% DMSO/25% 2‐hydroxypropyl‐*β*‐cyclodextrin (Sigma‐Aldrich, 332,607) in water (w/v). The pharmacokinetic study was carried on 40 male C57BL/6N mice (WTLH‐BJ) with a body weight average 40 g (*n* = 4 per group, 6–9 weeks). Animals were weighed prior to dose administration and dose volume was determined based on the body weights. The vehicle was 2% DMSO/25% of 2‐hydroxypropyl‐*β*‐cyclodextrin. Mice were orally administered by gavage with a single dose of 20 mg/Kg of the UB‐SCG‐51. Blood collection (about 0.03 mL per time point) was performed from saphenous vein of each animal into prechilled EDTA‐K2 tubes and placed on wet ice until centrifugation. Blood samples were centrifuged at 3200*g* for 10 min to obtain plasma, then transferred into 96‐well plates or polypropylene tubes followed by quick‐frozen over dry ice. A total quantity of 4 μL of plasma samples, standard, or control was added to 96‐well plate followed by quenching with 120 μL of Internal Standard (IS1). Brain tissue was homogenized using MeOH/15 mM PBS buffer at the ratio of 1:4 (1 g tissue with 4 mL buffer). A total quantity of 40 μL of tissue samples, standard, or control was added to 96‐well plate and quenched with 800 μL of IS1. and then each sample was vortex‐mixed for 10 min at 800 rpm. The samples were centrifuged for 15 min at 3220*g* and 50 μL of supernatant was directly injected for LC–MS/MS analysis. Mass spectra were recorded on a LC–MS/MS BI Triple Quad 6500 plus (Sciex) using a UPLC HSS T3 Column (ACQUITY) with column temperature at 45°C. Sample solutions (4 μL each) were injected applying a flow rate of 0.65 mL/min. IS1 is consists of six different mixture of material including Labetalol and tolbutamide and Verapamil and dexamethasone and glyburide and Celecoxib 100 ng/mL in ACN.

#### 2.4.1 Behavioral and cognitive tests

##### 2.4.1.1 Open field test (OFT)

The OFT was performed as previously described by Vasilopoulou et al.^28^ which evaluates anxiety‐like behavior. Mice were placed at the center of a white polywood box (50 × 50 × 25 cm) and allowed to explore it for 5 min. Behavior was scored with SMART® ver.3.0 software, and each trial was recorded for later analysis. The parameters scored included center staying duration, rearing, grooming, and the distance traveled.

##### 2.4.1.2 Object location test (OLT)

The OLT is a well‐established task to evaluate mice spatial performance.^29^ The test was carried out for 3 days in a wooden box (50 × 50 × 25 cm), in which three walls were white except one that was black. On the first day, the box was empty, and the animals just habituated to the open field arena for 10 min. On the second day, two objects were placed in front of the black wall, equidistant from each other and the wall. The objects were 10 cm high and identical. The animals were placed into the open field arena and allowed to explore both objects and surroundings, for 10 min. Afterward, the animals were returned to their home cages, and the OLT apparatus was cleaned with 70% ethanol. On the third day, one object was moved in front of the white wall to test the spatial memory. Trials were recorded using a camera mounted above the open field area, and the total exploration time was determined by scoring the amount of time (seconds) spent sniffing the object in the new location (TN) and the object in the old location (TO). To evaluate the cognitive performance, the DI was calculated, which is defined as (TN − TO)/(TN + TO).

##### 2.4.1.3 Novel object recognition test (NORT)

Using NORT, short‐ and long‐term recognition memory was analyzed. The test was conducted in a 90°, two‐arm, 25‐cm‐long, 20‐cm‐high maze. Light intensity in the middle of the field was 30 lux. The objects to be discriminated were plastic figures (object A, 5.25‐cm‐high, and object B, 4.75‐cm‐high). First, mice were individually habituated to the apparatus for 10 min for 3 days. On day 4, they were submitted to a 10‐min acquisition trial (first trial), during which they were placed in the maze in the presence of two identical novel objects (A + A or B + B) placed at the end of each arm. A 10‐min retention trial (second trial) occurred 2 h later. During this second trial, objects A and B were placed in the maze, and the times that the animal took to explore the new object (TN) and the old object (TO) were recorded. A Discrimination index (DI) was defined as (TN − TO)/(TN + TO). To avoid object preference biases, objects A and B were counterbalanced so that one half of the animals in each experimental group were first exposed to object A and then to object B, whereas the other one half first saw object B and then object A was presented. The maze, the surface, and the objects were cleaned with 70% ethanol between the animals' trials to eliminate olfactory cues.

#### 2.4.2 Biochemical experiments

##### 2.4.2.1 Brain isolation and immunoassays

Mice were killed 1 day after the last trial was conducted, and the brain was quickly removed from the skull. Hippocampus was dissected and frozen in powdered dry ice and maintained at −80°C for further use. Tissue samples were homogenized in lysis buffer containing phosphatase and protease inhibitors (Cocktail II, Sigma), and protein concentration was determined by the Bradford method. A total quantity of 20 μg of protein was separated by Sodium dodecyl sulfate‐Polyacrylamide gel electrophoresis (SDS‐PAGE) (8%–15%) and transferred onto Polyvinylidene difluoride (PVDF) membranes (Millipore). The membranes were blocked in 5% bovine serum albumin (BSA) in Tris‐buffered saline containing 0.1% Tween 20 (TBS‐T; Sigma) for 1 h at room temperature, followed by overnight incubation at 4°C with primary antibodies diluted in TBS‐T and 5% BSA (Sigma). See details in resource and reagent Table (Table S1). Then, membranes were washed and incubated with secondary antibodies for 1 h at room temperature. Immunoreactive proteins were visualized utilizing an enhanced chemiluminescence‐based detection kit (ECL kit; Millipore) and digital images were acquired employing an Imager 680 (Amersham Bioscience). Band intensities were quantified by densitometric analysis using Image Lab software (BioRad), and values were normalized to glyceraldehyde 3‐phosphate dehydrogenase (GAPDH).

For immunohistochemical and Thioflavin‐S staining studies, the frozen brains were embedded in OCT Cryostat Embedding Compound (Tissue‐Tek), cut into 30‐μm‐thick sections on a cryostat (Leyca Microsystems) at −20°C, and placed on slides. For Aβ plaques staining by thioflavin S, slides were rehydrated with phosphate‐buffered saline (PBS) for 5 min. Later, the brain sections were incubated with 0.3% Thioflavin‐S (Sigma‐Aldrich) for 20 min at room temperature in the dark. Subsequently, these were submitted to washes in 3‐min series, specifically with 80% ethanol (two washes), 90% ethanol (one wash), and PBS (three washes). Finally, the slides were mounted using Fluoromount‐G™ (EMS), allowed to dry overnight at room temperature in the dark, and then stored at 4°C, up to image acquisition.

Immunohistochemical studies started with 3 h of drying time at room temperature; then, slices were fixed with acetone at 4°C for 10 min, allowed to dry overnight, and finally stored at −20°C until their further staining. For the staining procedure, free‐floating slices were placed in a 24‐well plate and rehydrated by 5 min incubation in PBS. Afterward, the blocking/permeabilization step was performed (20 min in PBS‐BSA 1% + 1% Triton). Following two, 5 min washings in PBS, the slices were incubated overnight at 4°C with the primary antibodies (resource and reagent Table). Two further washings were carried out before incubation with the fluorescent secondary antibody (1 h at room temperature, (see resource and reagent Table). Finally, before mounting with Fluoromount‐G™ (EMS), nuclear staining was performed with Hoechst 33258 2 μg/mL (Sigma‐Aldrich) for 5 min at room temperature. Slides were allowed to dry overnight after mounting and image acquisition was performed with a fluorescence laser microscope (Olympus BX41) (10X objective). Images were acquired, maintaining constant exposure for all samples across single experiments. In this analysis, the number of the positive cells was measured in hippocampal *cornu ammonis* (CA) 1, CA3, and *dentate gyrus* (DG) areas, based on a group of stained pixels surrounded by unstained pixels. Thus, average size was calculated as the stained area of the positive cells divided by the total area unstained, representing the average size of the stained area. At least four images from four different individuals were analyzed with ImageJ/Fiji software available online from the National Institutes of Health). For plaque quantification, similar and comparable histological areas were selected, focusing on having the hippocampus and the whole cortical area positioned adjacently.

##### 2.4.2.2 Amyloid‐β protein level quantification by ELISA

We have used the amyloid‐Aβ_40_ ELISA kit (Invitrogen #KHB3481; ThermoFisher) and the amyloid‐Aβ_42_ ultrasensitive ELISA kit (Invitrogen, #KHB3441) to analyze the Aβ_40_ and Aβ_42_ protein levels, respectively. All procedures followed the manufacturer’s instructions.

##### 2.4.2.3 Western blotting

Microglia or astrocyte proteins were extracted by RIPA buffer (Thermo Fisher Scientific Inc.). Extracted proteins were separated by SDS/PAGE and subsequently transferred to nitrocellulose membranes (Bio‐Rad). Membranes were blocked in 3% BSA for 1 h at RT and incubated with primary antibodies (details in resource and reagent Table) overnight at 4°C, followed by incubation with Highly Cross‐Adsorbed Secondary Antibody, Alexa Fluor Plus 800 or 680 (Life Technologies) for 1 h at RT. Membranes visualized on Odyssey (LI‐COR Biosciences). Antibodies used in this work are listed in Table S1.

##### 2.4.2.4 RNA extraction and gene expression determination

Microglia or astrocyte total RNAs were isolated by using a Quick‐RNA kit (Zymo Research, Inc.). The concentration of total RNAs was measured using a UV–Vis spectrophotometer (NanoDrop8000, Thermo Fisher Scientific Inc.) and reverse‐transcribed with a high‐capacity cDNA reverse transcription kit (Applied Biosystems). Gene expression was quantified by Fast SYBR green real‐time PCR on a Quantstudio 5 system (Applied Biosystems). The primer sequences are listed in Table S1. Data were analyzed according to the comparative Ct method. *Gapdh* was used to normalize the amounts of cDNA within each sample.

Mice brain total RNA isolation was carried out by means of Trizol reagent following the manufacturer’s instructions. RNA content in the samples was measured at 260 nm, and sample purity was determined by the A260/280 ratio in a NanoDrop™ ND‐1000 (Thermo Scientific Inc). Samples were also tested in an Agilent 2100B Bioanalyzer (Agilent Technologies) to determine the RNA integrity number. Reverse transcription‐Polymerase chain reaction (RT‐PCR) was performed as follows: 2 μg of messenger RNA (mRNA) was reverse‐transcribed using the High Capacity (complementary DNA) cDNA Reverse Transcription kit (Applied Biosystems).

Real‐time PCR was performed on the Step One Plus Detection System (Applied Biosystems) employing the SYBR Green PCR Master Mix (Applied Biosystems). Each reaction mixture contained 6.75 μL of cDNA, whose concentration was 2 μg, 0.75 μL of each primer (whose concentration was 100 nM), and 6.75 μL of SYBR Green PCR Master Mix (2X). Data were analyzed utilizing the comparative cycle threshold (Ct) method (ΔΔCt), where the *actin* transcript level was utilized to normalize differences in sample loading and preparation. Each sample (*n* = 4) was analyzed in triplicate, and the results represented the *n*‐fold difference in transcript levels among different samples.

##### 2.4.2.5 RNA‐sequencing

Pools of four hippocampal tissue samples from the 5XFAD control and 5XFAD UB‐SCG‐51 mice (5 mg/Kg) were extracted using Trizol reagent (Thermo Fisher) and the purity and concentration was evaluated using NanoDrop One/One^C^ spectrophotometer (Thermo Fisher). The sample quality was assessed using Agilent Bioanalyzer 2100 and sequencing libraries were prepared using the RNA total amount of 5 μg. After QC confirmation, RNA library preparation was performed using TruSeq RNA Sample Prep Kit (Poly A capture). The samples were aligned with the reference genome using Bowtie2, and the gene expression level was estimated using RSEM. Differentially expressed genes were identified using the edgeR program. Genes showing altered expression with *p* < 0.05 and more than 1.5‐fold changes were considered differentially expressed. KEGG, Gene Ontology, and GSEA were used to perform the enrichment and pathway analysis using Enrichr database. Raw data were deposited at the Gene Expression Omnibus (accession GSE189250), raw fastq files for RNA‐seq on mouse hippocampus 5XFAD (accession GSE189249).

### 2.5 Statistical analysis

Data are expressed as the mean ± Standard error of the mean (SEM) from at least 14 individuals for behavioral tasks and 4–5 samples for molecular techniques. Besides, group size may vary according to power statistical analysis and expertise of the authors regarding the behavioral tests^30^ he blinded analysis was performed for behavioral tests. Likewise, we performed the Shapiro–Wilk test to verify data normality for all groups. Data analysis was conducted using GraphPad Prism version 9.2 statistical software. Means were compared with the one‐way‐ANOVA and post hoc analysis. Statistical significance was considered when *p* values were <0.05. Furthermore, statistical outliers were carried out with Grubbs' test and were removed from the analysis.

## 3 RESULTS

### 3.1 The sEHi, UB‐SCG‐51, inhibited inflammation and reactive conversion in primary glial cells

**FIGURE 1:**
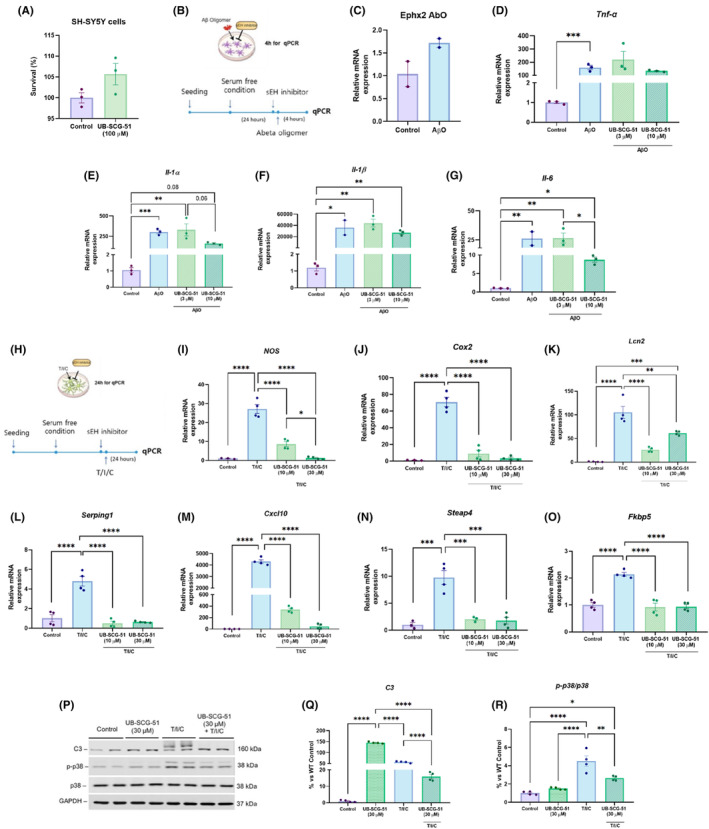
Reduction of inflammation markers after sEHi treatment in microglia and primary astrocyte cells. (A) Viability of SH‐SY5Y cells after 24 h exposure to UB‐SCG‐51 (100 μM). (B) Schematic diagram showing the AβO(Aβ_1–42_)‐treated microglia with UB‐SCG‐51. (C) *Ephx2* mRNA levels of AβO‐treated primary microglia compared to nonactivated microglia. (D–G) Representative gene expression levels of pro‐inflammatory markers in primary microglia: (D) *Tnf‐α*, (E) *Il‐1α*, (F) *Il‐1β*, and (G) *Il‐6*. (H) Schematic diagram showing the T/I/C‐treated astrocyte with UB‐SCG‐51. (I–O) Representative gene expression of reactive astrocytes markers: (I) *NOS*, (G) *Cox2*, (K) *Lcn2*, (L) *Serping1*, (M) *Cxcl10*, (N) *Steap4*, and (O) *Fkbp5*. (P) Immunoblot and quantification of (Q) C3, and (R) p‐p38/p38 ratio levels in reactive astrocyte. Values presented are the mean ± SEM. Groups were compared by Student *t*‐test and one‐way ANOVA and post hoc Tukey's test; [*n* = 2–4 per group (Outliers: *n* = 1 AbO in *Il‐1β* gene expression levels)]; *p < 0.05; **p < 0.01; ***p < 0.001; and ****p < 0.0001.

We first studied the effect of sEH inhibition in SH‐SY5Y cells exposed to UB‐SCG‐51 (100 μM in 24 h), and cells did not show any neuronal cell toxicity (Figure 1A). Furthermore, we studied the effect of sEH inhibition in primary glial cells (microglia and astroglia) exposed to UB‐SCG‐51 at different concentrations (30, 50, and 100 μM in 24 h), and there was no microglia cell toxicity at any concentration, whereas astrocyte cells showed 40% of toxicity only at high concentration of 100 μM. Then, to validate the sEHi efficacy in AβO‐induced microglial activation, mouse primary microglial cells isolated from CD1 brain tissue were pretreated with UB‐SCG‐51 followed by Aβ_1‐42_ (2 μM) (Figure 1B). On the one hand, AβO caused an increase in *Ephx2* gene expression in microglia compared to the control cells (Figure 1C). Likewise, AβO promoted and increased protein levels of EPHX2 in human astrocytes and mouse primary microglia, which was partially reverted after treatment with UB‐SCG‐51 at a concentration of 30 μM (Figure S2). Furthermore, AβO significantly induced mRNA expression for pro‐inflammatory cytokines, including *tumor necrosis factor α* (*Tnf‐α*), *interleukin‐1α* (*Il‐1α*), *interleukin‐1* (*Il‐1β*), and *interleukin‐6* (*Il‐6*), which were prevented by UB‐SCG‐51 at 10 μM concentration, but not at 3 μM (Figure 1D–G). Recently, it has been shown that activation of microglia leads to the conversion of normal astrocytes to reactive astrocytes via secretion of IL‐1α, TNF‐α and complement component 1 (C1q) (T/I/C) in a variety of neurodegenerative diseases including AD and Parkinson's disease.^31^ Human primary astrocytes treated with T/I/C for 24 h in the presence or absence of UB‐SCG‐51 (10 and 30 μM concentration) (Figure 1H) were used to investigate the effect of sEHi on reactive astrocyte conversion. UB‐SCG‐51 prevented the induction of potent inflammatory mediators, such as *nitric oxide synthase* (*NOS*) and *cyclooxygenase‐2* (*Cox2*) mRNAs after T/I/C (Figure 1G–I). Moreover, mRNA levels of reactive astrocyte representative markers, *Lipocalin 2* (*Lcn2*), *Serping1*, *C‐X‐C motif chemokine ligand 10* (*Cxcl10*), *Steap4 metalloreductase* (*Steap 4*), *FK506 binding protein 5* (*Fkbp5*), and *complement 3* (*C3*), reactive astrocyte representative markers were significantly reduced after UB‐SCG‐51 treatment (Figure 1K–O). Consistent with the inhibitory effect of UB‐SCG‐51 in mRNA analysis, protein levels of C3, and phosphor‐p38 were decreased in T/I/C‐induced reactive astrocyte treated with UB‐SCG‐51 (Figure 1P–R).

### 3.2 Pharmacokinetic properties and ability to cross the BBB of UB‐SCG‐51

**FIGURE 2:**
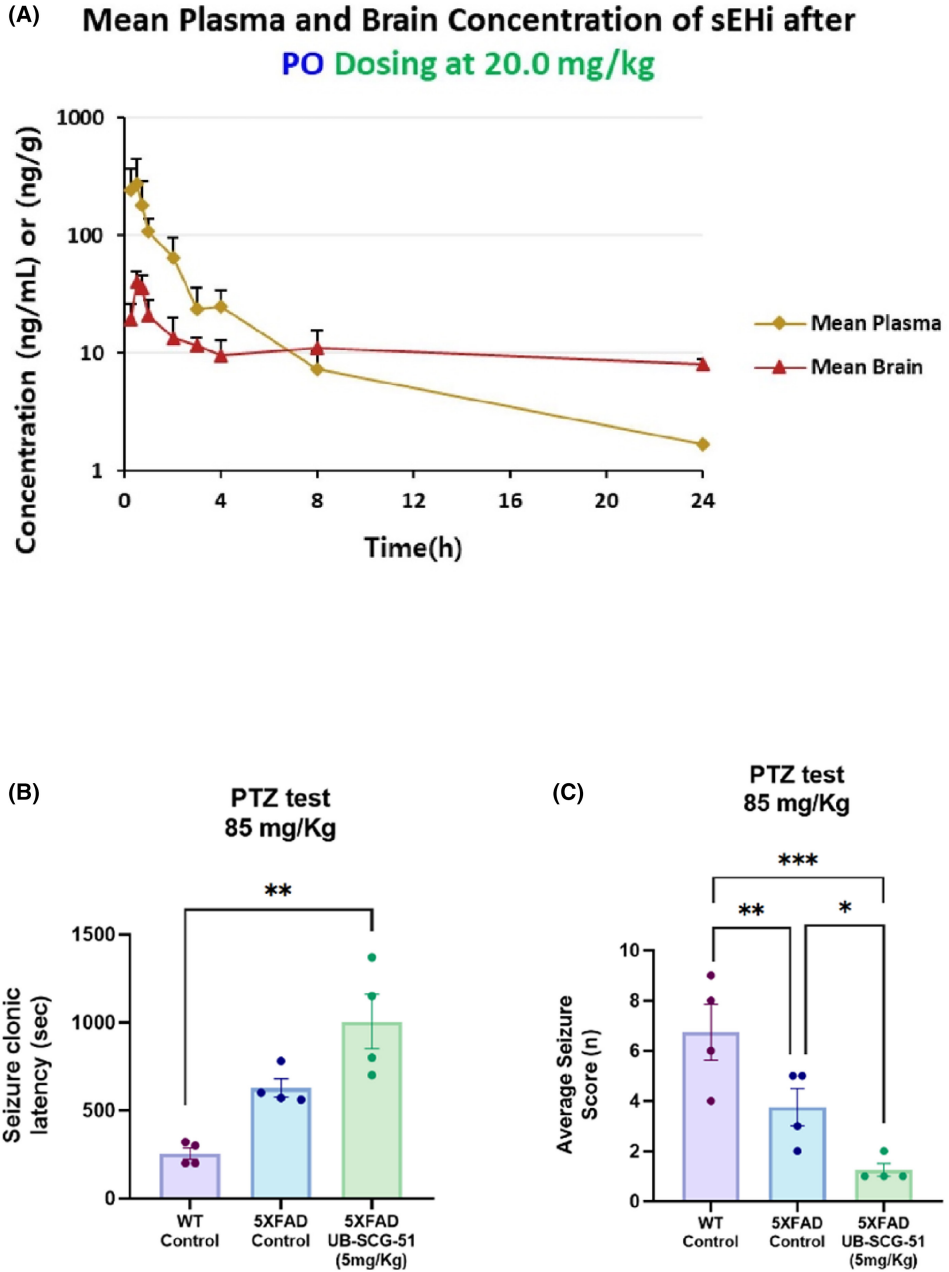
Pharmacokinetic properties and ability to cross the BBB of UB‐SCG‐51. (A) Mean plasma and brain concentration of UB‐SCG‐51 after IV dosing at 20 mg/Kg. (B, C) PTZ test: (B) Quantification of seizure clonic latency. (C) Representation of average seizure score. Values presented are the mean ± SEM. Groups were compared by one‐way ANOVA and post hoc Tukey's test; (*n* = 4 per group); *p < 0.05; **p < 0.01, and ***p < 0.001.

To characterize the pharmacokinetic profile of UB‐SCG‐51 after oral administration, the compound levels were measured in the plasma brain of mice (Figure 2A and Table S3). Following a single oral administration of UB‐SCG‐51 (20 mg/Kg), the absorption of UB‐SCG‐51 from the gastrointestinal tract was fast, reaching a maximum plasma concentration (C_max_) of 278 ± 169 ng/mL after 0.5 h. Interestingly, sufficient concentrations of UB‐SCG‐51 were found in the brain, indicating efficient blood–brain barrier (BBB) penetration. Concretely, a C_max_ 41 ± 7.99 ng/mL was determined in the brain, which was reached after about 0.5 h (T_max_).

Moreover, we performed the pentylenetetrazol (PTZ) test to investigate the ability of UB‐SCG‐51 to cross the blood–brain barrier (BBB). Then, a standard acute test involving the administration of pro‐convulsant PTZ was employed.^32^, ^33^ Furthermore, the PTZ assay is considered highly translatable from mice to humans. In the test, PTZ was administered at 85 mg/Kg by the subcutaneous route and the time to onset of first clonic seizure, an average of clonic seizures, tonic seizure latency and lethality were monitored for 30 min. Finally, vehicles or sEH inhibitors (TPPU and UB‐SCG‐51) were administered by gavage at 5 mg/Kg 1 h prior to pro‐convulsant. Interestingly, TPPU, and the compound UB‐SCG‐51 treatment at 5 mg/Kg delay the onset of tonic seizures induced by PTZ in comparison with the control group (vehicle) (Table S4). Note that animals that did not display tonic seizure within 30 min were excluded from the table. Strikingly, the average seizure score was significantly lower in the UB‐SCG‐51 group than the others two groups (Figure 2C). Besides, the TPPU‐treated group showed a significant reduction in comparison with the control group (Figure 2C). Altogether, we showed that our compound UB‐SCG‐51 protects mice from convulsions and associated lethality, demonstrating that this compound can cross the BBB.

### 3.3 sEHi rescued cognitive impairment in 5XFAD mice

**FIGURE 3:**
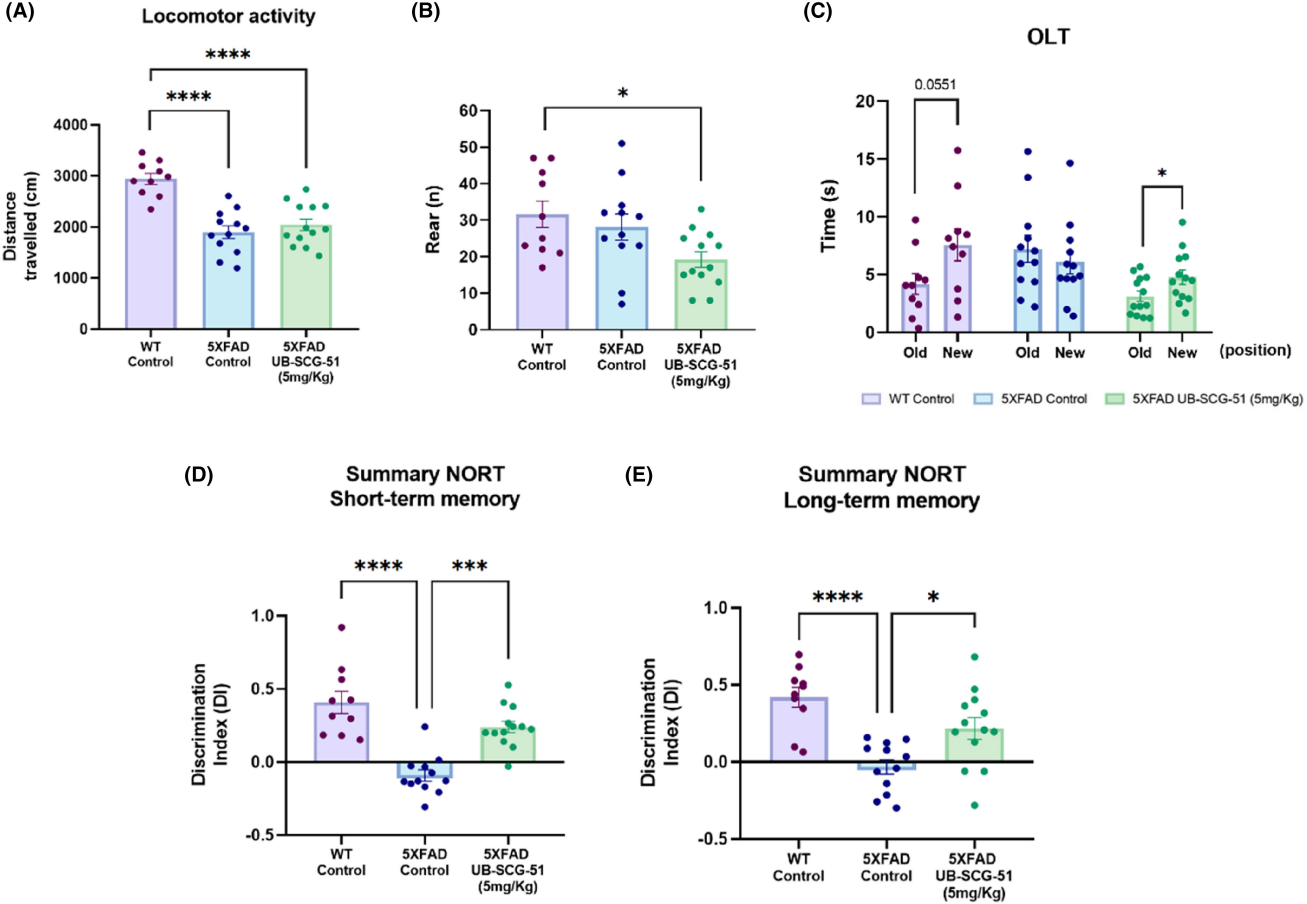
Cognitive status recovered after sEHi treatment in 5XFAD mice model. (A, B) For OFT: (A) Locomotor activity and (B) number of rearings (C) For OLT: Time spend on old and new object. (D, E) For NORT: (D) Short‐term memory test at 2 h and (E) long‐term memory test at 24 h. Values presented are the mean ± SEM. Groups were compared by one‐way ANOVA test and post hoc Tukey's test; (*n* = 10–13 per group); *p < 0.05; ***p < 0.001; and ****p < 0.0001.

Amyloid pathology is associated with cognitive and synaptic impairment. Therefore, we performed cognitive studies to examine whether UB‐SCG‐51 could rescue them in 5XFAD mice, a well‐established AD model to explore the neuropathological mechanisms of cognitive impairment.^34^ Therefore, we evaluated the anxiety‐like behavior using OFT. Our results revealed that the UB‐SCG‐51 treatment did not substantially modify the 5XFAD behavioral treats, such as locomotor activity that was usually diminished compared to the WT mice group (Figure 3A). However, the number of rears was reduced in the UB‐SCG‐51‐treated mice group, reaching significance compared to the WT Ct group (Figure 3B).

The OLT evaluation showed the cognitive deficits associated with spatial memory previously described in 5XFAD.^29^ No differences were determined between the time depicted in the novel object location and the old position in contrast with the WT Ct group (Figure 3C). Remarkably, the results allowed us to demonstrate the neuroprotective effects of UB‐SCG‐51 treatment in 5XFAD mice, increasing in a significant way the time exploration of the new location in comparison with the old location (Figure 3C), indicating a spatial memory rescue.

To evaluate working memory, the mice were assessed in the NORT. 5XFAD UB‐SCG‐51 group exhibited significantly improved memory capabilities, obtaining the highest DI compared to the 5XFAD Ct values, both at short‐ and long‐memory paradigms (Figure 3D,E). Thus, our data showed that sEH inhibition prevented cognitive impairment in 5XFAD mice.

### 3.4 sEH inhibition boosted Aβ degradation, reduced plaque deposition, and decreased tau hyperphosphorylation in 5XFAD mice

**FIGURE 4:**
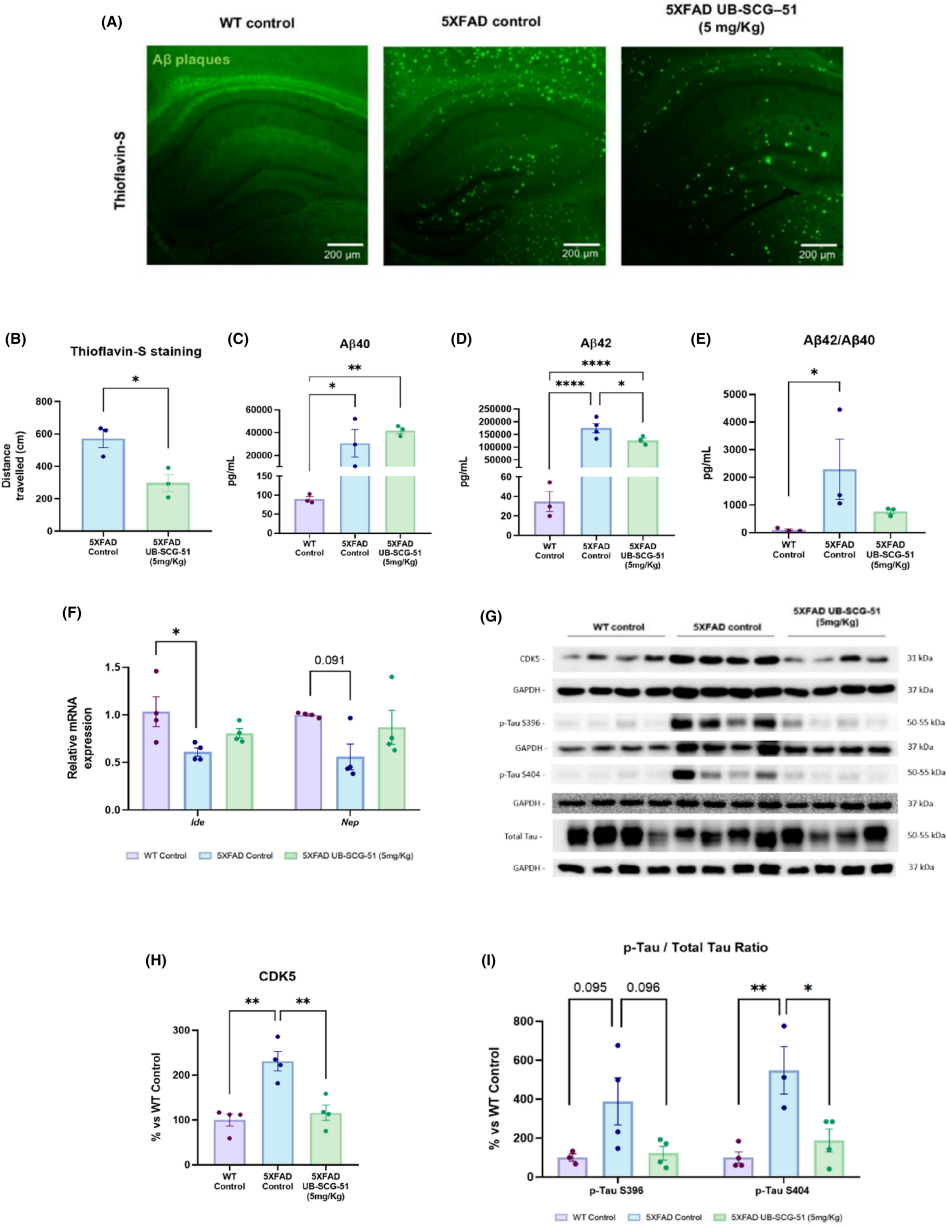
Reduction of AD hallmarks after treatment. (A) Representative images of Aβ plaques stained with Thioflavin‐S in the hippocampus of WT Ct, 5XFAD Ct, and 5XFAD UB‐SCG‐51 mice. (B) Quantitative analysis of Thioflavin‐S staining. (C) Quantification of total Aβ_40_ protein, (D) total Aβ_42_ protein, and (E) Aβ_42_/Aβ_40_ ratio. (F) Representative gene expression levels of *Ide* and *Nep*. (G) Immunoblots and representative quantification of (H) CDK5, and (I) *p*‐Tau S396/total Tau and *p*‐Tau S404/total Tau ratios. Values presented are the mean ± SEM. Groups were compared by Student *t*‐test and one‐way ANOVA and post hoc Tukey's test; (*n* = 3 per group in A–E, and *n* = 4 per group F–I (Outliers: *n* = 1 in 5XFAD Control in *p*‐Tau S404 ratio levels); *p < 0.05; **p < 0.01; and ****p < 0.0001.

AD is characterized by two neuropathological marks: extracellular Aβ plaque deposits and NFTs of Tau protein.^35^ Of note, 5XFAD mice have both Aβ and *p*‐Tau aggregates.^36^ Thus, the number of Aβ plaques in 5XFAD was evaluated by histochemical Thioflavin‐S staining. As expected, WT mice's brains did not stain with Thioflavin‐S, but 5XFAD mice presented an important number of Aβ plaques (Figure 4A,B). Interestingly, the number of Aβ plaques was significantly lower in the brain of 5XFAD treated with UB‐SCG‐51 (Figure 4A,B), indicating the neuroprotective role after sEH inhibition. As expected, a significant increase in Aβ_40_, Aβ_42_ levels (Figure 3C,D), and in the Aβ_42_/Aβ_40_ ratio were found in 5XFAD Ct mice in comparison with the WT group (Figure 4E). More importantly, UB‐SCG‐51 significantly reduced the Aβ_42_ levels and the Aβ_42_/Aβ_40_ ratio, whereas Aβ_40_ did not reach significance (Figure 4C–E). Moreover, *insulin‐degrading enzyme* (*Ide*) and *neprilysin* (*Nep*) expression, amyloid degrading enzymes decreased in 5XFAD Ct mice compared to the WT Ct group (Figure 4F). Interestingly, UB‐SCG‐51 treatment slightly increased *Ide* and *Nep* expression in 5XFAD compared to the 5XFAD Ct group (Figure 4F).

As mentioned above, *p*‐Tau is another important AD hallmark in the 5XFAD mice model.^28^ Tau phosphorylation is modulated by several protein kinases such as cyclin‐dependent kinase 5 (CDK5),^37^ which promotes *p*‐Tau and NFTs formation.^38^ CDK5 and *p*‐Tau at S404 and S396 were evaluated by WB (Figure 4G–I). First, UB‐SCG‐51 decreased CDK5 protein levels in the 5XFAD‐treated group, similar to WT Ct (Figure 4G,H). Accordingly, phosphorylated and total Tau were higher in the 5XFAD Ct group compared to the WT Ct. Besides, the UB‐SCG‐51‐treated mice significantly reduced *p*‐Tau S396 and *p*‐Tau S202 ratios (Figure 4G–I). Collectively, these results highlighted the beneficial effects of sEHi, reducing AD hallmarks.

### 3.5 UB‐SCG‐51 treatment reduced glial cells reactivity and pro‐inflammatory markers

**FIGURE 5:**
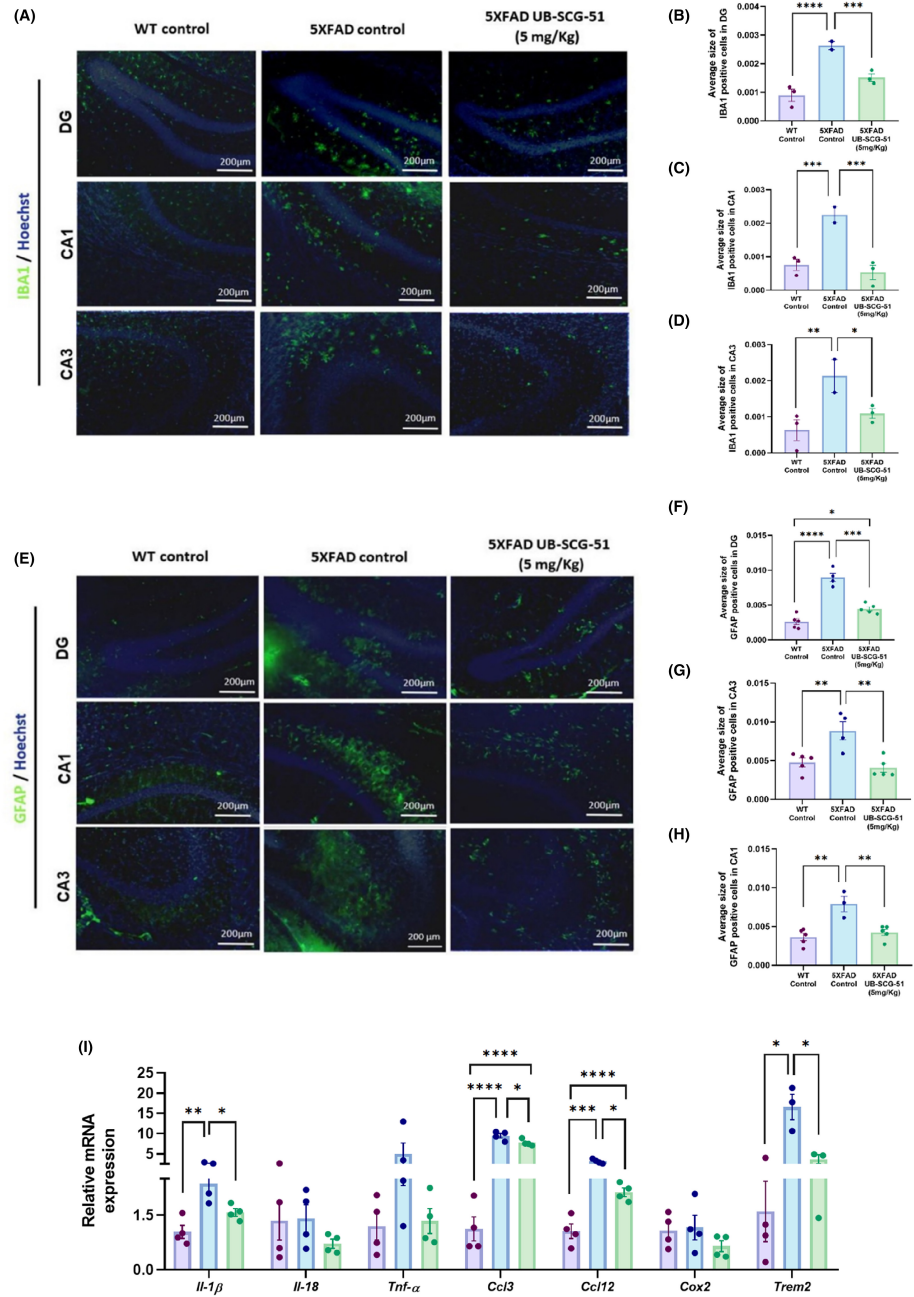
(A) Representative images for Iba‐1 immunostaining and its relative expression quantification in (B) *dentate gyrus* (DG), (C) *cornu ammonis* (CA) 1, and (D) CA3. (E) Representative images for GFAP immunostaining and its relative expression quantification in (F) DG, (G) CA1, and (H) CA3. (I) Representative gene expression levels of inflammatory markers *for Il‐1β*, *Il‐18*, *Tnf‐α*, *Ccl3*, *Ccl12*, *Cox2*, and *Trem2*. Values presented are the mean ± SEM. Groups were compared by one‐way ANOVA and post hoc Tukey's test; (*n* = 3–4 per group (Outliers: 1 in 5XFAD Control, 1 in 5XFAD treated in *Trem2* gene expression levels); *p < 0.05; **p < 0.01; ***p < 0.001; and ****p < 0.0001.

Activation of microglia and astrogliosis plays an important role in the development and progression of AD.^39^ Also, it is well documented that microglial activation is increased in 5XFAD mice as well as astrogliosis.^40^ Then, we assessed ionized calcium‐binding adaptor molecule 1 (Iba‐1) by IHC experiments to evaluate changes in microglia reactivity. Remarkably, UB‐SCG‐51 treatment significantly reduced Iba‐1 levels in DG, CA1, and CA3 hippocampal areas compared to the 5XFAD Ct group (Figure 5A–D). Importantly, UB‐SCG‐51 treatment also attenuated astrogliosis in the hippocampus of 5XFAD brains by significantly decreasing the Glial Fibrillary Acidic Protein (GFAP) immunoreactivity in DG, CA1, and CA3 areas in comparison with untreated 5XFAD mice (Figure 5E–H).

Finally, we also evaluated gene expression of pro‐inflammatory cytokines associated with microglial activation, such as interleukin‐1β (*Il‐1β*), interleukin‐18 (*Il‐18*), Tnf‐α, C‐C chemokine ligand 3 (*Ccl3*), C‐C Chemokine ligand 12 (*Ccl12*), cyclooxygenase‐2 (*Cox2*), and triggering receptor expressed on myeloid cells 2 (*Trem2*). Those pro‐inflammatory markers revealed a higher expression in 5XFAD compared to the WT Ct group (Figure 5I). Interestingly, *Il‐1β*, *Ccl3*, *Ccl12*, and *Trem2* were significantly lower in 5XFAD treated with UB‐SCG‐51 compared to the 5XFAD Ct group (Figure 5I). Collectively, those results demonstrated that UB‐SCG‐51 protects against glial activation in 5XFAD mice.

### 3.6 sEH inhibition with UB‐SCG‐51 induces a transcriptional profile that benefits on cognitive performance

**FIGURE 6:**
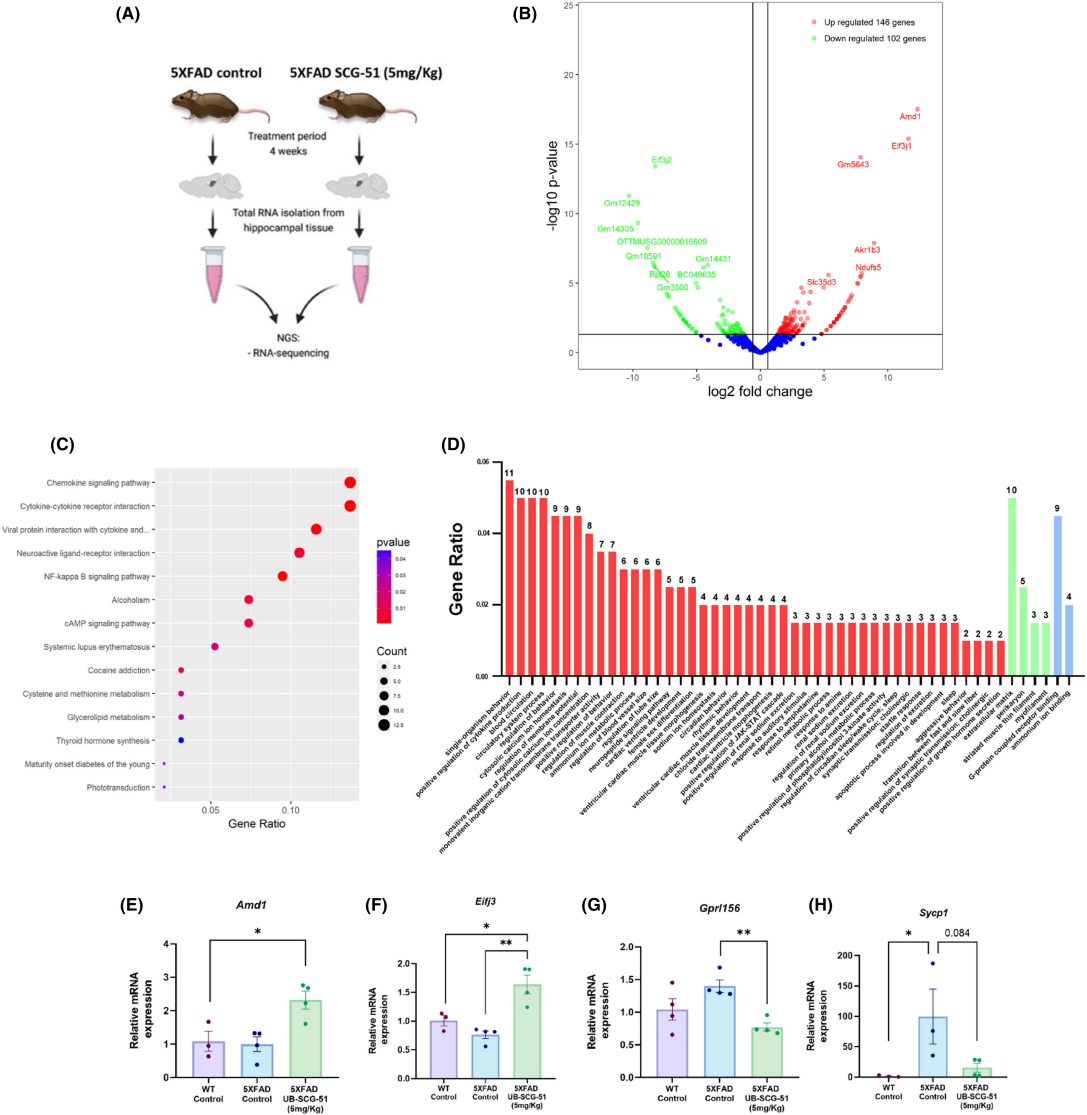
Transcriptomic modifications after UB‐SCG‐51 treatment. (A) Schematic representation of RNA‐sequencing process. (B) Volcano plot analysis. (C). KEGG pathway enrichment after UB‐SCG‐51 treatment. (D) GO process enriched after UB‐SCG‐51 treatment. (E–H) For qPCR validation genes: (E) *Amd1*, (F) *Eif3j*, (G) *Gpr156*, and (H) *Sycp1* gene expression levels. Values presented are the mean ± SEM. Groups were compared by one‐way ANOVA and post hoc Tukey's test; (*n* = 4 per group (Outliers: *n* = 1 WT control in *Amd1* and *Eif3j* gene expression levels; *n* = 1 5XFAD control, and one in 5XFAD treated in *Sycp1* gene expression levels); *p < 0.05; **p < 0.01.

To characterize the transcriptional profile associated with UB‐SCG‐51 treatment, we evaluated the transcriptome by RNA‐seq from 5XFAD Ct and 5XFAD treated with UB‐SCG‐51 (Figure 6A). Differential expression analysis identified 248 differentially expressed genes (DEG; fold change cutoff ≥ 1.5, *p* <0.05) in 5XFAD treated with UB‐SCG‐51 compared with 5XFAD Ct. Concretely, we found 146 genes increased and 102 reduced (Figure 6B and Table S5).

Functional analysis showed that the differentially expressed genes (DEGs) regulate processes such as the chemokine signaling pathway, cytokine–cytokine receptor interaction, and neuroactive ligand–receptor interaction (Figure 6C,D). These processes are important in regulating the inflammation and plasticity of neuronal networks in the central nervous system.^41^, ^42^ Strikingly, we found an increase in the transcripts associated with neuronal plasticity, which allows us to explain, in part, the neuroprotective effects on cognition promoted by UB‐SCG‐51 in the 5XFAD mice. On the other hand, we identified a reduction in transcripts linked to the nuclear factor‐kappa B (NF‐κB) signaling pathway (Figure 6C,D), suggesting the implication of sEH inhibition reducing neuroinflammation in this early onset Alzheimer disease (EOAD) mouse model.

To validate the RNA‐seq, we performed RT‐qPCR for some DEGs of interest, such as *Adenosylmethionine decarboxylase 1* (*Amd1*), *Eukaryotic translation Initiation factor 3 subunit J* (*Eif3j*), *G Protein‐coupled receptor* (*Gpr156*), and *Synaptonemal complex protein 1* (*Sycp1*), which were significantly deregulated after UB‐SCG‐51‐treated 5XFAD compared to 5XFAD Ct (Figure 5E–H). These data suggest that treatment with UB‐SCG‐51 promotes a transcriptional profile that benefits on cognition and neuronal plasticity.

### 3.7 sEH inhibition treatment fostered the eIF2α/CHOP pathway, triggering a response to endoplasmic reticulum stress in 5XFAD mice

**FIGURE 7:**
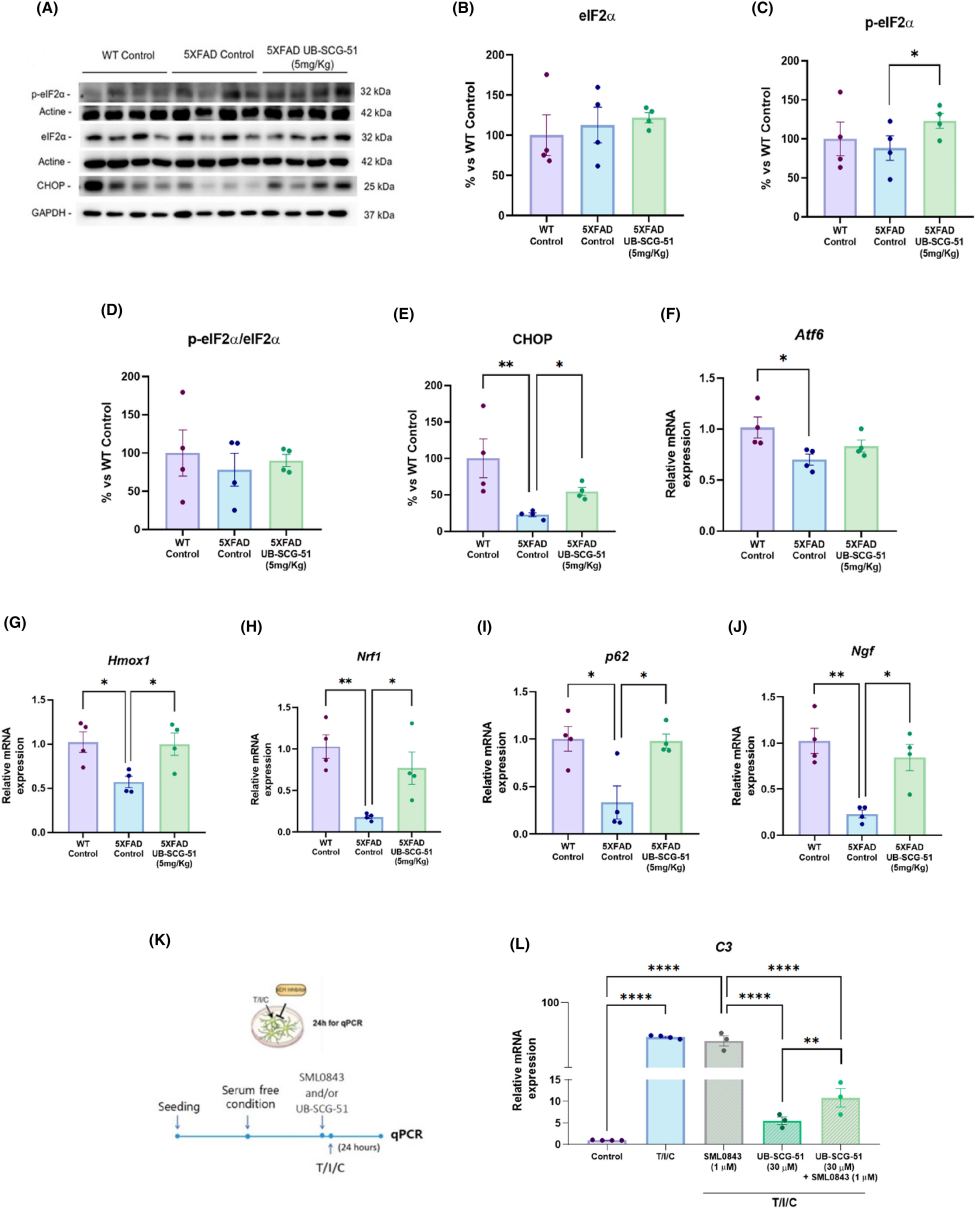
eIF2α pathway activation after sEHi treatment. (A) Immunoblots and representative quantification of (B) eiF2α, (C) p‐eiF2α, (D) the ratio of p‐eiF2α/ eiF2α, and (E) CHOP. (F) Representative gene expression levels of *Atf6*, (G) *Hmox‐1*, (H) *Nrf1*, (I) *p62*, and (J) *Ngf*. (K) Schematic diagram showing the T/I/C‐treated astrocyte with UB‐SCG‐51 and/or SML0843. (L) Representative gene expression levels of *C3*. Values presented are the mean ± SEM. Groups were compared by one‐way ANOVA and post hoc Tukey's test; (*n* = 4 per group, *n* = 3–4 per group in cell culture experiments); *p < 0.05; **p < 0.01; and ****p < 0.0001.

As shown, *Eif3j* gene expression increase was found in RNA‐seq (Figure 6F). Importantly, eIF3 interacts with eIF2 generating pre‐initiation ternary complexes to initiate protein synthesis.^43^ This eukaryotic initiation factor 2α (eIF2α) globally controls protein synthesis in several tissues, regulating gene expression.^44^ These clues prompted us to study one of the most harmful signaling regulated by de novo protein synthesis in neurological diseases such as AD: the unfolded protein response (UPR).^44^, ^45^ In addition, it has been observed that UPR interacts with the autophagy proteolytic system, and together, they play an important role in survival processes during stress.^45^ Remarkably, we found a significant increase of *p*‐eIF2α levels in 5XFAD treated with UB‐SCG‐51 compared to the 5XFAD Ct group (Figure 7A–D). Likewise, we found a higher *p*‐eIF2α/eIF2α ratio in the 5XFAD‐treated group compared to the 5XFAD Ct, indicating the activation of the eIF2α signaling pathway (Figure 7A–D).^43^, ^45^, ^46^

Then, we evaluated the C/EBP homologous protein (CHOP), a master coordinator of the integrated stress response (ISR) and the autophagy activation gene transcriptional program.^47^ Interestingly, CHOP protein levels were increased in 5XFAD treated with UB‐SCG‐51 compared to the 5XFAD Ct (Figure 7E). In addition, we evaluated the gene expression of *Activating transcription factor 6*, also known as *Atf6*, which is involved in the UPR and induces CHOP.^48^ Remarkably, we found a reduction in Atf6 gene expression in 5XFAD Ct compared to the WT Ct group, but no changes between both 5XFAD were found (Figure 7F). Considering these results, we evaluated *heme oxygenase 1* (*Hmox‐1*), *nf‐e2‐related factor 1* (*Nrf1*), *ubiquitin‐binding protein p62* (*p62*), and *nerve growth factor* (*Ngf*), which are genes that induce and modulate autophagy processes.^45^, ^49^, ^50^ Accordingly, we found an increase of these genes in 5XFAD UB‐SCG‐51‐treated mice compared to the 5XFAD Ct, suggesting an enhancement of the autophagy process (Figure 7F–I).

Finally, we demonstrated that eIF2α/CHOP signaling pathway is involved in the neuroprotective effects promoted by sEHi. We evaluated in human primary astrocytes, the C3 protein levels, an inflammatory marker. We confirmed that it was increased in primary astrocytes activated by T/I/C combination (Figures 1Q, 7J,K). Interestingly, eIF2α inhibitor (eIF2αi, SML0843) did not modify the *C3* gene expression (Figure 7J,K). Furthermore, a significant reduction in *C3* gene expression after UB‐SCG‐51 single treatment was found (Figure 7J,K). Strikingly, SML0843 co‐treatment with sEHi was able to partially block the UB‐SCG‐51 *C3* gene expression increase, indicating the participation of the eIF2α/CHOP signaling pathway in the neuroprotective effects promoted by sEH inhibition.

### 3.8 UB‐SCG‐51 treatment reduced Calpain/Caspase signal pathway in 5XFAD mice

**FIGURE 8:**
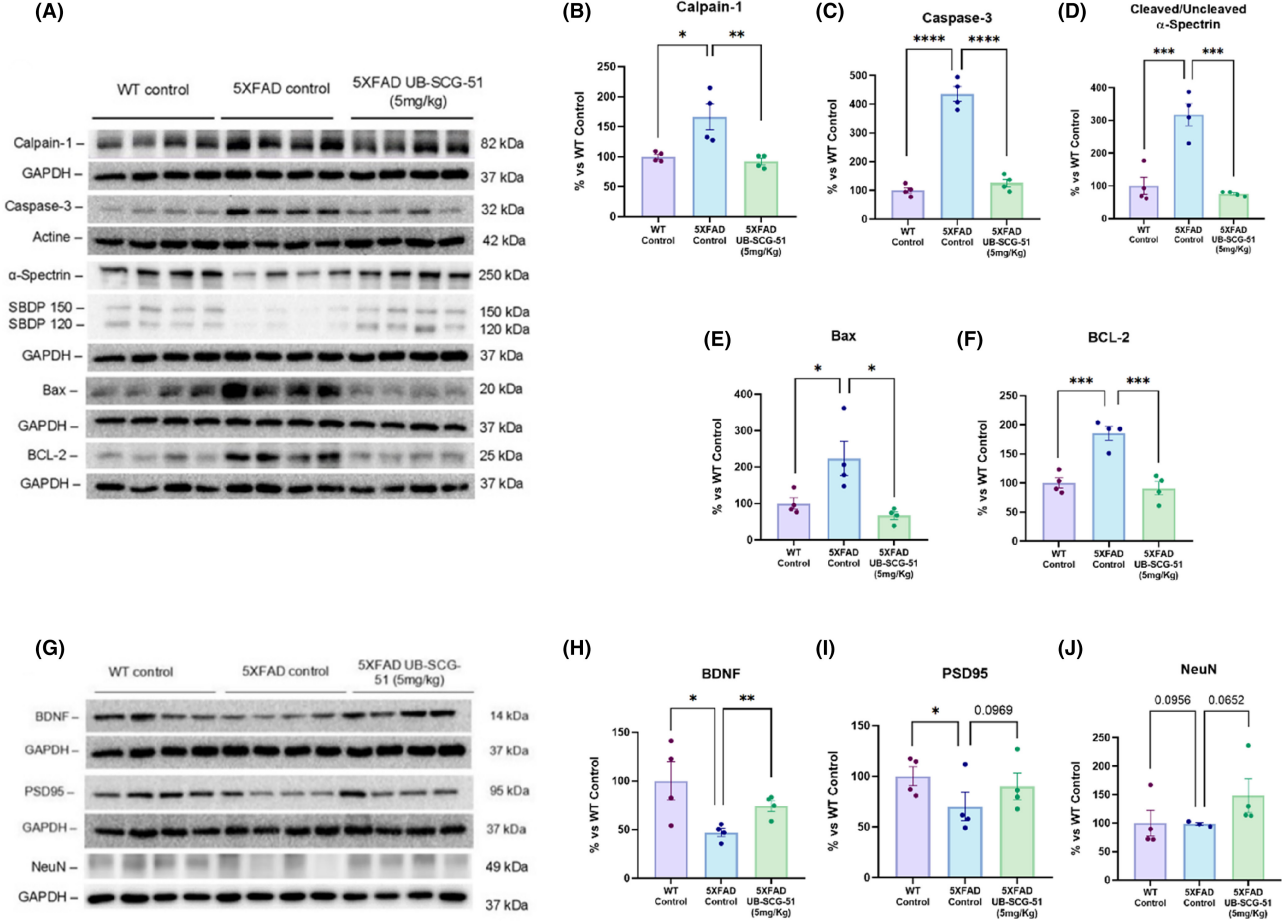
Reduction of apoptosis and increase of synaptic markers after sEHi treatment. (A–F) For apoptotic markers: (A) Immunoblot and quantification of (B) Calpain‐1, (C) Caspase‐3, (D) Uncleaved/cleaved α‐spectrin ratio, (E) Bax, and (F) Bcl‐2. (G–J) For synaptic markers: (G) Immunoblot and quantification of (H) BDNF, (I) PSD95, and (J) NeuN. Values presented are the mean ± SEM. Groups were compared by one‐way ANOVA and post hoc Tukey's test; (*n* = 4 per group (Outliers: *n* = 1 in 5XFAD control in NeuN protein levels); *p < 0.05; **p < 0.01, ***p < 0.001; and ****p < 0.0001.

The Calpain/Caspase pathway has been implicated in neuronal death following neurodegenerative diseases such as AD.^51^ Here, we hypothesized that sEHi with UB‐SGC‐51 might play a role in modulating mitochondria‐induced apoptosis via modifying the levels of some B‐cell lymphoma 2 (Bcl‐2) family proteins. Accordingly, Calpain‐1 and Caspase‐3 protein levels were higher in 5XFAD than in Wt mice (Figure 8A–C). Furthermore, Caspase‐3 activation was demonstrated through *α*‐spectrin breakdown products (SBDPs). Particularly, results showed that 5XFAD Ct mice presented the highest cleaved/uncleaved SBDPs ratio compared to the WT Ct, indicating activation of Caspase‐3 (Figure 8A,D). Likewise, our results showed that UB‐SCG‐51 treatment significantly reduced the levels of both proteins in 5XFAD‐treated mice, reducing their activity (Figure 8A–C). Finally, the evaluation of Bcl‐2‐associated X (Bax) and Bcl‐2 protein levels revealed that both were increased in 5XFAD Ct mice compared to the WT Ct, and that treatment with UB‐SCG‐51 delivered WT mice levels (Figure 8A,E,F), indicating that Calpain/Caspase activity was reduced after sEH inhibition.

Moreover, pathologically, it is well described that 5XFAD presents an impairment in synaptic plasticity and neuronal death in the hippocampal brain area.^34^ Here, we found a significant increase in brain‐derived neurotrophic factor (BDNF) protein levels in the 5XFAD treated with UB‐SCG‐51 compared to the 5XFAD Ct group (Figure 8G,H). Furthermore, as an outcome of synaptic plasticity and neuronal loss, we evaluated the postsynaptic density protein 95 (PSD95) and neuronal nuclear protein (NeuN) protein levels. Consequently, we found a clear improvement in PSD95 and NeuN protein levels in 5XFAD treated with UB‐SCG‐51 compared to the 5XFAD Ct group but did not reach statistical significance (Figure 8G,I,J). Thus, our data confirmed that sEH inhibition rescued synaptic weakening and neuronal loss in 5XFAD mice.

## 4 DISCUSSION

Up to now, all approved AD therapies do not target the main hallmarks of the disease. Unfortunately, these treatments are symptomatic and not disease‐modifying therapies, which ultimately leads to limited effects in terms of clinical benefit.^52^ Unlike current treatments for AD, pharmacological inhibition of sEH focuses on reducing the neurodegenerative processes that trigger cognitive decline, neuroinflammation and, eventually, the clinical manifestations of dementia.^23^, ^53^ This study addressed the anti‐inflammatory effects linked to sEH inhibition by using a new and potent sEHi, UB‐SCG‐51,^27^ in primary glial cell cultures and 5XFAD mice. However, since AD is multifactorial, it should be noted that there are other important players in the neurodegenerative landscape besides neuroinflammation such as amyloid‐β accumulation, Tau pathology, oxidative stress, and neuronal loss. Therefore, we delved into neuroprotective molecular pathways undescribed after sEH inhibition by evaluating transcriptional changes and validating them in in vitro and in vivo models. In this regard, we unraveled new molecular pathways based on UPR, ERS, and apoptosis modulation. The study's findings corroborated our premise related to the contribution of sEH to cognitive impairment and AD.

The presence of microglia around amyloid plaques has mainly been described in humans^54^ and rodent models.^55^ Here, we promoted microglial activation in vitro by oligomeric Aβ, and we monitored it through the mRNA levels of *Ephx2*, *Il‐1α*, *Tnf‐α*, *Il‐1β*, and *Il‐6*. Of note, UB‐SCG‐51 treatment significantly prevented the increase found in these genes expression. Current reports described that activated microglia convert astrocytes to neurotoxic reactive astrocytes by releasing TNF‐α, IL‐1α, and C1q (T/I/C). In our hands, UB‐SCG‐51 potently reduced most of the reactive astrocyte markers (*Nos*, *Cox2*, *Lcn2*, *Serping1*, *Cxcl10*, *Steap4*, *Fkbp5*, and *C3*) in T/I/C‐induced reactive astrocytes. In addition, we found an unexpected result related to increased levels of C3 in sEHi only‐treated group. However, the classical complement cascade was downregulated also in this only sEHi‐treated group, confirming the neuroprotective effects. Interestingly, C3 is also a critical mediator of synaptic refinement and plasticity,^56^ thereby could be the explanation for these increased levels after sEHi treatment in healthy conditions. Thus, collectively, these data support previous studies, suggesting that the therapeutic effect of sEH inhibition is likely due to the blockade of neuroinflammation induced by reactive astrocytes.^19^, ^23^, ^57^

The 5XFAD is a well‐established double transgenic APP/PSEN1 mouse model for AD. It co‐expresses five familial AD mutations and incorporates AD pathological characteristics, including early Aβ plaques formation, gliosis, and robust cognitive and behavioral alterations such as memory impairment.^30^, ^58^, ^59^, ^60^, ^61^ First, from in vivo experiment, we showed an enhanced oral bioavailability and immediate brain disposition when applied by oral gavage administration of the UB‐SCG‐51 at a 20 mg/Kg dose, indicating that it can cross the BBB efficiently and reach the target in the brain in a sufficient concentration. Furthermore, we demonstrated that UB‐SCG‐51 administered orally to 5XFAD at 7 months of age significantly rescued the working and spatial memory loss, without any effect on locomotor activity, reinforcing previous reports describing the beneficial effects of sEHi on different rodent models of neurodegeneration.^23^, ^24^, ^25^

Regarding the neuroinflammatory in vivo modulation after sEH inhibition, as expected, we corroborated an increase in microgliosis and astrogliosis in 5XFAD mice, measured by Iba‐1 and GFAP immunostaining.^62^, ^63^ However, Lee et al.^25^ reported that astrocytes and pro‐inflammatory cytokines were increased in the brain of sEH‐depleted APP/PS1 mice. This may be because sEH was knocked out and, in our case, we are making a pharmacological inhibition that allows, even in a lesser amount, to carry out the function of sEH, either referred to inflammatory functions or other molecular pathways. Consequently, higher levels of several pro‐inflammatory cytokines derived from glial cells were found. Interestingly, *Il‐1β*, *Il‐18*, *Ccl3*, *Ccl12*, *and Trem2* were significantly reduced after UB‐SCG‐51 treatment, supporting the reduction in astrogliosis and microgliosis through in vitro assays. Of note, this is consistent with previous reports on the feasibility of sEH inhibition to face neurodegenerative diseases^64^, ^65^ and AD.^19^, ^66^ In addition to gliosis reduction, we reported Aβ plaques and *p*‐Tau diminution in EOAD and late‐onset Alzheimer disease (LOAD) mice models after sEHi treatment, pinpointing the ability of sEHi to modulate the two major AD hallmarks.^23^ Accordingly, UB‐SGC‐51 boosted Aβ degradation, reducing the number of Aβ plaques in the 5XFAD brain. Remarkably, after treatment with UB‐SCG‐51, we showed about a 26% increase in the levels of Aβ_40,_ an amyloid fragment that inhibits Aβ accumulation,^67^ indicating a putative mechanism by which sEHi reduced Aβ burden accumulation in 5XFAD. In the same way, we observed about 37% reduction in Aβ_42_ levels after sEHi treatment, being the Aβ_42_/Aβ_40_ ratio significantly reduced after treatment, as has been described previously for other sEH.^23^ The aberrant Tau hyperphosphorylation characteristic of AD was also prevented by UB‐SCG‐51 treatment. Specifically, we demonstrated a reduction in Tau kinase CDK5 and several Tau phospho‐epitopes, S396 and S404, giving further support to the neuroprotective effects of sEHi. Therefore, results reinforced the role of sEH dysfunction in the etiopathogenesis of the disease and that treatment with sEHi rescued neuronal impairment associated with AD hallmarks by reducing glial activation.

After validating the beneficial effects of the sEHi UB‐SCG‐51, our next goal was to go deeper into the molecular pathways implicated in the beneficial effects of sEH inhibition in an AD mice model with an RNA‐seq approach. Its analysis rendered important transcriptional changes after UB‐SCG‐51 treatment, indicating the influence of sEH activity on the transcriptomic profile of 5XFAD mice. From transcriptomic analysis, the KEGG enrichment analysis revealed the association of sEH with neuroinflammation, regulation of behavior, and synaptic transmission, among others. One of the most important upregulated signaling pathways in UB‐SCG‐51 5XFAD‐treated mice was eIF2α, validated by qPCR (*Eif3j* gene). It is known that p‐eIF2α reduces protein synthesis but increases the transcription of specific genes in response to stressors.^68^, ^69^, ^70^ Furthermore, one of the first reactions to cope with ERS is suppressing general protein synthesis mediated by eIF2α phosphorylation.^71^ The rest of UPR signaling activates transcription cascades to synthesize selective sets of proteins that can promote protein folding and increase the degradation of unfolded/misfolded ER protein.^72^ In addition, p‐eIF2α/CHOP signaling modulates the cell adaptation via translational control.^73^ Hence, to the best of our knowledge, here we demonstrated for the first time the induction of the eIF2α signaling pathway in the 5XFAD brain after the inhibition of sEH, highlighting this pathway as a master key to explaining therapeutic expectations for sEHi. Moreover, *Atf6* gene expression levels were not modified in the 5XFAD‐treated group with UB‐SCG‐51, indicating no implication of this transcription factor in the neuroprotection promoted by UB‐SCG‐51. Then, to pave this hypothesis, CHOP protein levels were found higher after treatment with UB‐SCG‐51, as well as the *Nrf*, *p62*, and *Ngf* gene expression involved in autophagy processes.^45^, ^49^, ^50^ Likewise, *Hmox1* gene expression, a known regulator of inflammation^74^ and UPR pathway^75^, ^76^ was increased after sEHi treatment. Notably, higher basal levels of *Hmox1* were associated with reduced responses to the inflammatory environment,^77^ suggesting that the sEH inhibition approach could promote a dual action, directly modulating neuroinflammation and increasing the response of other molecular pathways such as UPR signaling.

Then, given that C3 is the central component of the complement system, which is activated by the ERS^78^ and is highly expressed in AD,^79^ being associated with pathological Aβ aggregation, we evaluated *C3* gene expression levels in T/I/C‐activated astrocytes. *C3* gene expression in activated astrocytes was about 10x more than the control cells and was completely blocked by UB‐SCG‐51 treatment. However, eIF2α inhibition by SML0843 did not affect *C3* gene overexpression in T/I/C‐activated astrocytes. Supporting our mechanistic hypothesis, SML0843 was able to reduce the beneficial effects of UB‐SCG‐51 because in co‐treatment *C3* gene expression levels were 2x fold in T/I/C‐activated astrocytes. These results demonstrated the participation of the eIF2α/CHOP signaling pathway in the neuroinflammatory inhibition promoted by sEH inhibition. Therefore, with all results presented thus far, it is feasible to suggest that sEH inhibition allows the activation of eIF2α, promoting a dual action via UPR signaling.

Finally, it has been described that accumulating misfolded proteins in the ER can overwhelm the protein quality control, causing cell death in AD.^80^ Then, cells can activate the UPR, which mediates cell survival by slowing protein translation^81^ and being proapoptotic Bcl‐2 proteins overexpressed, leading to the activation of the Caspase cascade, and generating their related breakdown products of α‐spectrin such as SBDP 150 and 120.^76^ Furthermore, another main Bcl‐2 function is to restrict pro‐apoptotic Bax protein, preserving mitochondrial outer membrane integrity,^82^ due to this, as we found a reduction in Bax, the Bcl‐2 also were reduced in 5XFAD sEHi‐treated group. Thus, to corroborate that this UPR activation does not promote cell death, we investigated the Calpain/Caspase signaling in the 5XFAD mice model. Our experiments demonstrated that UB‐SCG‐51 treatment significantly inhibited Calpain‐1 and Caspase‐3, which was then confirmed by the cleaved/uncleaved SBDPs ratio reduction in the 5XFAD‐treated group, showing that sEHi treatment prevented an intrinsic apoptotic process in transgenic mice. Concomitantly, as aforementioned, a reduction of Bax and Bcl‐2 protein levels was also demonstrated in 5XFAD‐treated group. These results suggest that the elF2α/CHOP‐mediated UPR activation elicited a decrease in proteases implicated in neuronal damage and apoptosis, thus promoting neuroprotection. Lastly, we found a joint increase in BDNF, PSD95, and NeuN synaptic plasticity markers after UB‐SCG‐51, indicating better neuronal status and cognitive performance. This study also had some limitations. First, all in vivo analyses were conducted using female 5XFAD mice, which limits representation of other forms of AD and introduces sex bias. The 5XFAD model is a well‐established AD model, basically representing the amyloid‐β hypothesis^58^ and referring to the sex bias, it is well described that female 5XFAD mice had higher levels of human APP and amyloid‐β and heightened inflammation versus males,^83^ for those reasons, we used females instead males. However, additional preliminary experiments were done with males, showing the same pattern profile. In addition, one must also be cautious in directly extending conclusions drawn from results with isolated glial cells on mice models, although molecular results in those are according to the participation of eiF2α/CHOP in reducing neuroinflammation and apoptosis in mice.

## 5 CONCLUSIONS

**FIGURE 9:**
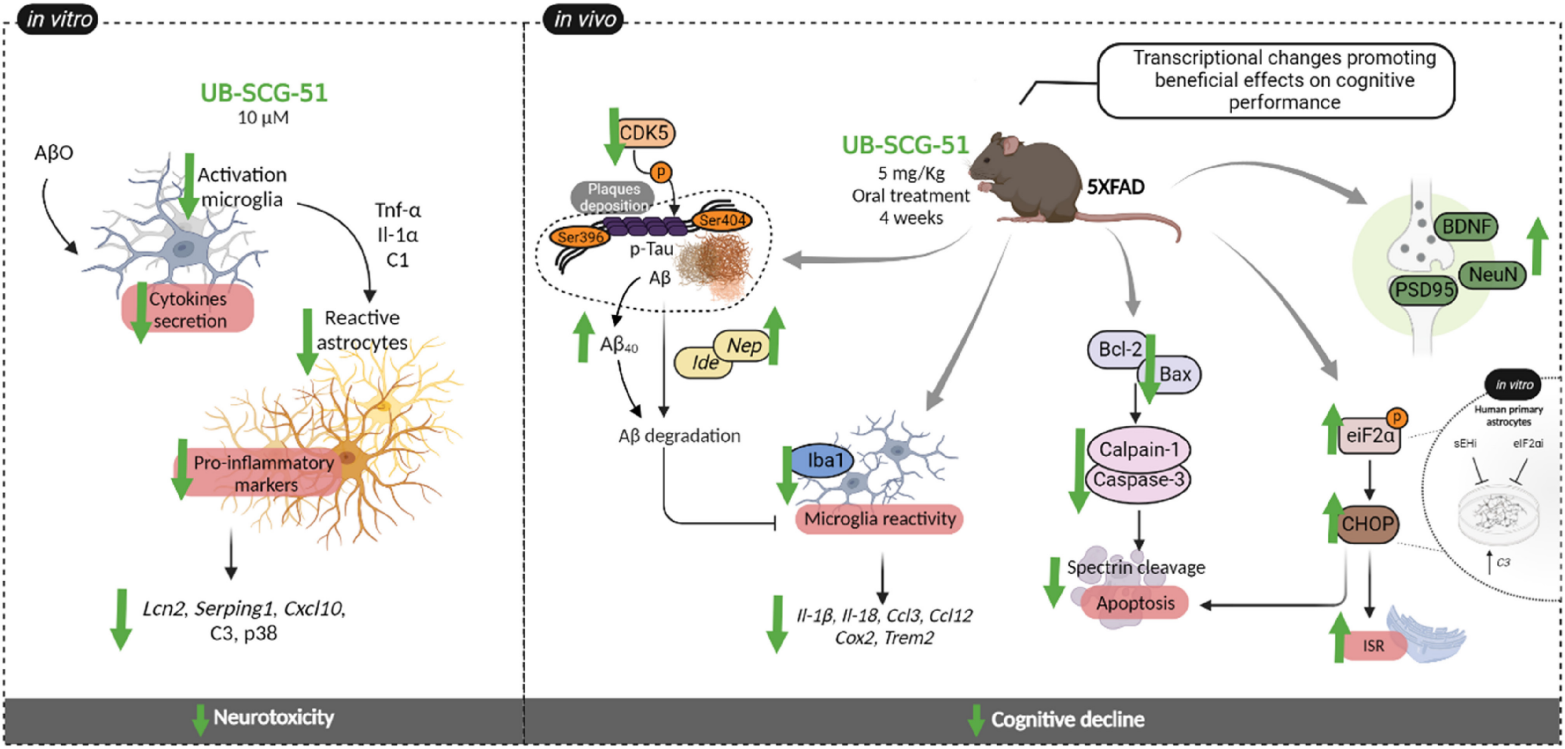
Illustrative scheme of the UB‐SCG‐51 effects in 5XFAD mice.

Based on these findings, we demonstrated that sEH inhibition reduced pro‐inflammatory processes in vitro and in vivo models, being the eIF2α/CHOP, an undescribed implicated neuroprotective pathway that collaborates improving cognition and AD hallmarks in 5XFAD mice model by favoring synaptic plasticity, modulating ISR, and apoptosis (Figure 9).

## AUTHOR CONTRIBUTIONS

CG‐F, MP: study conception and design; CG‐F, JJ‐F, DP‐I, AB‐S, SC, YO, and SL experimentation; CG‐F, MP data analysis, data interpretation, and article writing; SV: study conception and article writing. All authors read and approved the final article.

## FUNDING INFORMATION

This work was supported by Spanish Ministerio de Ciencia e Innovación (PID2019‐106285/AEI/10.13039/501100011033; PDC2021‐121096/AEI/10.13039/501100011033; María de Maeztu Unit of Excellence to Institute of Neurosciences, University of Barcelona, MDM‐2017‐0729) and 2021SGR357 (AGAUR, Catalonia). ABS acknowledges a FI SDUR (2021 FI‐B0812) fellowship to AGAUR Catalonia.

## CONFLICT OF INTEREST STATEMENT

The authors declare no competing financial interests.

## Supporting information


Appendix S1:

## Data Availability

Gene Expression Omnibus (accession GSE189250), raw fastq files for RNA‐seq on mouse hippocampus 5XFAD (accession GSE189249). This article does not report original code. Any additional information required to reanalyze the data reported in this article is available from the lead contact upon request.
