# Supplementary material for: Novel molecular mechanism driving neuroprotection after soluble epoxide hydrolase inhibition: Insights for Alzheimer's disease therapeutics
Source: CNS Neurosci Ther. 2023 Oct 31;30(4):e14511. doi: 10.1111/cns.14511 (PMC11017401; doi:10.1111/cns.14511)
Supplement: Supplementary file 1 — Appendix S1: [file CNS-30-e14511-s001.docx]

# Supplementary Table S1 | Reagents and Ressources

| REAGENT or RESOURCE | SOURCE | IDENTIFIER |
| --- | --- | --- |
| Antibodies |  |  |
| Goat-anti-mouse Alexa Fluor Plus 680 | Life Technologies | A32729 |
| Goat-anti-rabbit Alexa Fluor Plus 800 | Life Technologies | A32735 |
| Donkey-anti-rabbit Alexa Fluor 647 | Invitrogen^TM^ | AB253618 |
| EPHX2 | Abcam | AB155280 |
| GAPDH | Santa Cruz | SC-32233 |
| GFAP | Dako | AB10013382 |
| IBA1 | Abcam | AB2832244 |
| p-p38 | Santa Cruz | sc-166182 |
| p38 | Santa Cruz | sc-81621 |
| CDK5 | Santa Cruz | sc-173 |
| p-Tau (Ser396) | Invitrogen^TM^ | 44-752G |
| p-Tau (Ser404) | Invitrogen^TM^ | 44-758G |
| Tau Total | Invitrogen^TM^ | AHB0042 |
| C3 | R&D system | AF2655 |
| Calpain-1 | BioRad | AHP2443 |
| Caspase-3 | BD Transduction Lab | C31720 |
| α-spectrin | Millipore | MAB1622 |
| Bax | Cell Signalling | #2774 |
| Bcl-2 | Cell Signalling | #2870 |
| BDNF | Bios | BS-4989R |
| PSD95 | Abcam | AB18258 |
| GAPDH | Millipore | MAB374 |
| Actin | Invitrogen^TM^ | MA5-15739 |
| Donkey-anti-goat HRP conjugated | Santa Cruz | sc-2020 |
| Donkey-anti-rabbit HRP conjugated | BioRad | 170-6515 |
| Donkey-anti-mouse HRP conjugated | BioRad | 170-5047 |
| Reagents and drugs |  |  |
| UB-SCG-51 | This lab |  |
| p-eIF2α signal inhibitor | Sigma | SML0843 |
| IL-1α | Peprotech | 200-01A |
| TNF-α | R&D | 210-TA |
| C1q | R&D | 9134-TN |
| Astrocyte Medium-animal | Sciencell | #1831 |
| Astrocyte Medium-animal | Sciencell | #1801 |
| Bovine serum albumin (BSA) | Sigma | A7030 |
| Cell titer Glo | Promega | G7570 |
| Chemiluminescence-based detection kit | ECL Kit, Millipore | WBKLS0500 |
| Dulbecco’s modified Eagle’s medium (DMEM) | Gibco | 30-2002 |
| Eukitt® Quick-hardening mounting medium | Sigma-Aldrich | 25608-33-7 |
| Fetal bovine serum | Gibco | 30-2020 |
| Fluoromount-G™ | Thermo Fisher | 00-4958-02 |
| High Capacity cDNA Reverse Transcription kit | Applied Biosystems^TM^ | 4368814 |
| Hoechst 33258 solution | Sigma Aldrich | 33258 |
| Human Astrocytes medium | ScienCell | #1800 |
| Maxima SYBR Green qPCR Master Mix (2X), ROX solution provided | Thermo Scientific^TM^ | K0253 |
| Mowiol | Calbiochem | 9002-89-5 |
| Nitrocellulose membranes | BioRad | #1620150 |
| OCT Cryostat Embedding Compound | Scigen 4586 | 23-730.625 |
| Penicillin-streptomycin | Gibco | 1540122 |
| Phosphatase and protease inhibitors (Cocktail II) | Sigma | P5726 |
| Polyvinylidene difluoride (PVDF) membranes | Millipore | IPVH00010 |
| Quick-RNA kit | Zymo Research | R1055 |
| RIPA buffer | Thermo Scientific^TM^ | 89900 |
| Sodium dodecyl sulfate-Polyacrylamide gel electrophoresis (SDS-PAGE) (8‒15%) | Millipore | CAS 151-21-3 \| 817034 |
| TBS-T | This lab |  |
| Thioflavin S | Sigma Aldrich | 1326-12-1 |
| TRIsure^TM^ | Bioline, Meridian Bioscience | BIO-38032 |
| Critical commercial assays |  |  |
| Human amyloid-β_40_ ELISA Kit | Invitrogen^TM^ | KHB3481 |
| Human amyloid-β_42_ Ultrasensitive ELISA Kit | Invitrogen^TM^ | KHB3544 |
| Mouse Aβ_42_ ELISA Kit | Invitrogen^TM^ | KMB3441 |
| Mouse Aβ_40_ ELISA Kit | Invitrogen^TM^ | KMB3481 |
| Oligonucleotides |  |  |
| *Tnf-α* forward primer (5’-3’) TCGGGGTGATCGGTCCCCAA | This paper | Invitrogen |
| *Tnf-α* reverse primer (5’-3’) TGGTTTGCTACGACGTGGGCT | This paper | Invitrogen |
| *Il-1α* forward primer (5’-3’) GCACCTTACACCTACCAGAGT | This paper | Invitrogen |
| *Il-1α* reverse primer (5’-3’) AAACTTCTGCCTGACGAGCTT | This paper | Invitrogen |
| *Il-1β* forward primer (5’-3’) ACAGAATATCAACCAACAAGTGATATTCTC | This paper | Invitrogen |
| *Il-1β* reverse primer (5’-3’) GATTCTTTCCTTTGAGGCCCA | This paper | Invitrogen |
| *Il-6* forward primer (5’-3’) ATCCAGTTGCCTTCTTGGGACTGA | This paper | Invitrogen |
| *Il-6* reverse primer (5’-3’) TAAGCCTCCGACTTGTGAAGTGGT | This paper | Invitrogen |
| *NOS* forward primer (3’-5) GGCAGCCTGAGAGACCTTTG | This paper | Invitrogen |
| *NOS* reverse primer (5’-3’) GGAAGCGTTTCGGGATCTGAA | This paper | Invitrogen |
| *Cox2* forward primer (3’-5) TGACCCCCAAGGCTCAAATA | This paper | Invitrogen |
| *Cox2* reverse primer (5’-3’) CCCAGGTCCTCGCTTATGATC | This paper | Invitrogen |
| *Lcn2* forward primer (3’-5) CCAGTTCGCCATGGTATTTT | This paper | Invitrogen |
| *Lcn2* reverse primer (5’-3’) CACACTCACCACCCATTCAG | This paper | Invitrogen |
| *Serping1* forward primer (3’-5) ACAGCCCCCTCTGAATTCTT | This paper | Invitrogen |
| *Serping1* reverse primer (5’-3’) GGATGCTCTCCAAGTTGCTC | This paper | Invitrogen |
| *Cxcl10* forward primer (3’-5) GGCTAGTCCTAATTGCCCTTGG | This paper | Invitrogen |
| *Cxcl10* reverse primer (5’-3’) TTGTCTCAGGACCATGGCTTG | This paper | Invitrogen |
| *Steap4* forward primer (3’-5) CCCGAATCGTGTCTTTCCTA | This paper | Invitrogen |
| *Steap4* reverse primer (5’-3’) GGCCTGAGTAATGGTTGCAT | This paper | Invitrogen |
| *Fkbp5* forward primer (3’-5) TATGCTTATGGCTCGGCTGG | This paper | Invitrogen |
| *Fkbp5* reverse primer (5’-3’) CAGCCTTCCAGGTGGACTTT | This paper | Invitrogen |
| Atf6 forward primer (5’-3’) TTTCAGGGCAGGGCCATT | This paper | Invitrogen |
| Atf6 reverse primer (3’-5) CCCGGGACAAACAGGTCTT | This paper | Invitrogen |
| *Il-18* forward primer (3’-5) GTTTACAAGCATCCAGGCACAG | This paper | Invitrogen |
| *Il-18* reverse primer (5’-3’) GAAGGTTTGAGGCGGCTTTC | This paper | Invitrogen |
| *Ccl3* forward primer (3’-5) TGACCCCCAAGGCTCAAATA | This paper | Invitrogen |
| *Ccl3* reverse primer (5’-3’) CCCAGGTCCTCGCTTATGATC | This paper | Invitrogen |
| *Ccl12* forward primer (3’-5) ACACTGGTTCCTGACTCCTCT | This paper | Invitrogen |
| *Ccl12* reverse primer (5’-3’) ACCTGAGGACTGATGGTGGT | This paper | Invitrogen |
| *Trem2* forward primer (3’-5) CCTGAAGAAGCGGAATGGG | This paper | Invitrogen |
| *Trem2* reverse primer (5’-3’) CTTGATTCCTGGAGGTGCT | This paper | Invitrogen |
| *Ide* forward primer (3’-5) CCAAAAGGAAGCGTTCGCC | This paper | Invitrogen |
| *Ide* reverse primer (5’-3’) GGGATCGCTGATGAGAAGCA | This paper | Invitrogen |
| *Nep* forward primer (3’-5) TTGGGAGACCTGGCGGAAAC | This paper | Invitrogen |
| *Nep* reverse primer (5’-3’) CATTCCTTGGACCCTCACCCC | This paper | Invitrogen |
| *Amd1* forward primer (3’-5) AGCCATCAGTAGGGCTTCGT | This paper | Invitrogen |
| *Amd1* reverse primer (5’-3’) TACTAAGCTCCCACCCCGTT | This paper | Invitrogen |
| *Eif3j* forward primer (3’-5) TGGATCTTGTTGCCAAGGGG | This paper | Invitrogen |
| *Eif3j* reverse primer (5’-3’) TCATGTTGCAACCTTTGTTTCCA | This paper | Invitrogen |
| *Gpr156* forward primer (3’-5) GCCTGACCAACCATGTCAGC | This paper | Invitrogen |
| *Gpr156* reverse primer (5’-3’) GGAAGCTTTTGCTGGGAGTG | This paper | Invitrogen |
| *Sycp1* forward primer (3’-5) ACCGTTGGACAACGATTGCT | This paper | Invitrogen |
| *Sycp1* reverse primer (5’-3’) ATCCATTGCAAGTAAAAGCAACA | This paper | Invitrogen |
| *Hmox1 forward primer* (3’-5) TGACACCTGAGGTCAAGCAC | This paper | Invitrogen |
| *Hmox1 reverse primer* (5’-3’) GTCTCTGCAGGGGCAGTATC | This paper | Invitrogen |
| *Nrf1 forward primer* (3’-5) AGCACGGAGTGACCCAAAC | This paper | Invitrogen |
| Nrf1 reverse primer (5’-3’) TGTACGTGGCTACATGGACCT | This paper | Invitrogen |
| *p62 forward primer* (3’-5) CGTAAGGACCTTCTGGATCG | This paper | Invitrogen |
| p62 reverse primer (5’-3’) CGTCGTGGATGGTGAAATTG | This paper | Invitrogen |
| *Ngf forward primer* (3’-5) *GGAGCGCATCGAGTGACTT* | This paper | Invitrogen |
| Ngf reverse primer (5’-3’) CCTCACTGCGGCCAGTATAG | This paper | Invitrogen |
| *β-actin* forward primer (3’-5) CAACGAGCGGTTCCGAT | This paper | Invitrogen |
| *β-actin* reverse primer (5’-3’) GCCACAGGTTCCATACCCA | This paper | Invitrogen |
| Software and algorithms |  |  |
| EdgeR-package: Empirical analysis of digital gene expression data in R | Open source | https://rdrr.io/bioc/edgeR/man/edgeR-package.html |
| Enrichr database | Open source | http://amp.pharm.mssm.edu/Enrichr |
| Gene Ontology resource | Open source | http://geneontology.org/ |
| Graphpad Prism 9.2 | Graphpad Software | https://www.graphpad.com/ |
| GSEABase-package: Gene set enrichment data structures and methods | Open source | https://rdrr.io/bioc/GSEABase/man/GSEABase-package.html |
| ImageJ/Fiji | NIH | https://imagej.nih.gov/ij/download.html |
| KEGG PATHWAY Database | Open source | https://www.genome.jp/kegg/pathway.html |
| Deposited data |  |  |
| Gene Expression Omnibus | NCBI | accession GSE189250 |
| RNA-seq on mouse hippocampus 5XFAD | raw fastq files | accession GSE189249 |

**Supplementary Figure. S2 | a.** Representative images of human astrocytes at different conditions. **b.** Viability of primary microglia cells after 24h exposure to UB-SCG-51 at different concentrations. **c.** Viability of astroglia cells after 24h exposure to UB-SCG-51 at different concentrations. **d-e.** Immunoblot and quantification of human astrocytes were treated with UB-SCG-51 followed by TNF-α (3 0 ng/ml, R&D), IL-1α (3 ng/ml, Peprotech), and C1q (400 ng/ml, R&D) for 24h. **f-g.** Immunoblot and f. quantification of mouse primary microglia were treated with UB-SCG-51 followed by AβO (1 uM) for 4h. The cells were collected for further analysis of western blotting with anti-sEH (ab155280) antibody. Values presented are the mean ± SEM. Groups were compared by One-Way ANOVA and post-hoc Tukey’s test; (n = 3 per group); ***p*<0.01; ****p*<0.001.

**a.**


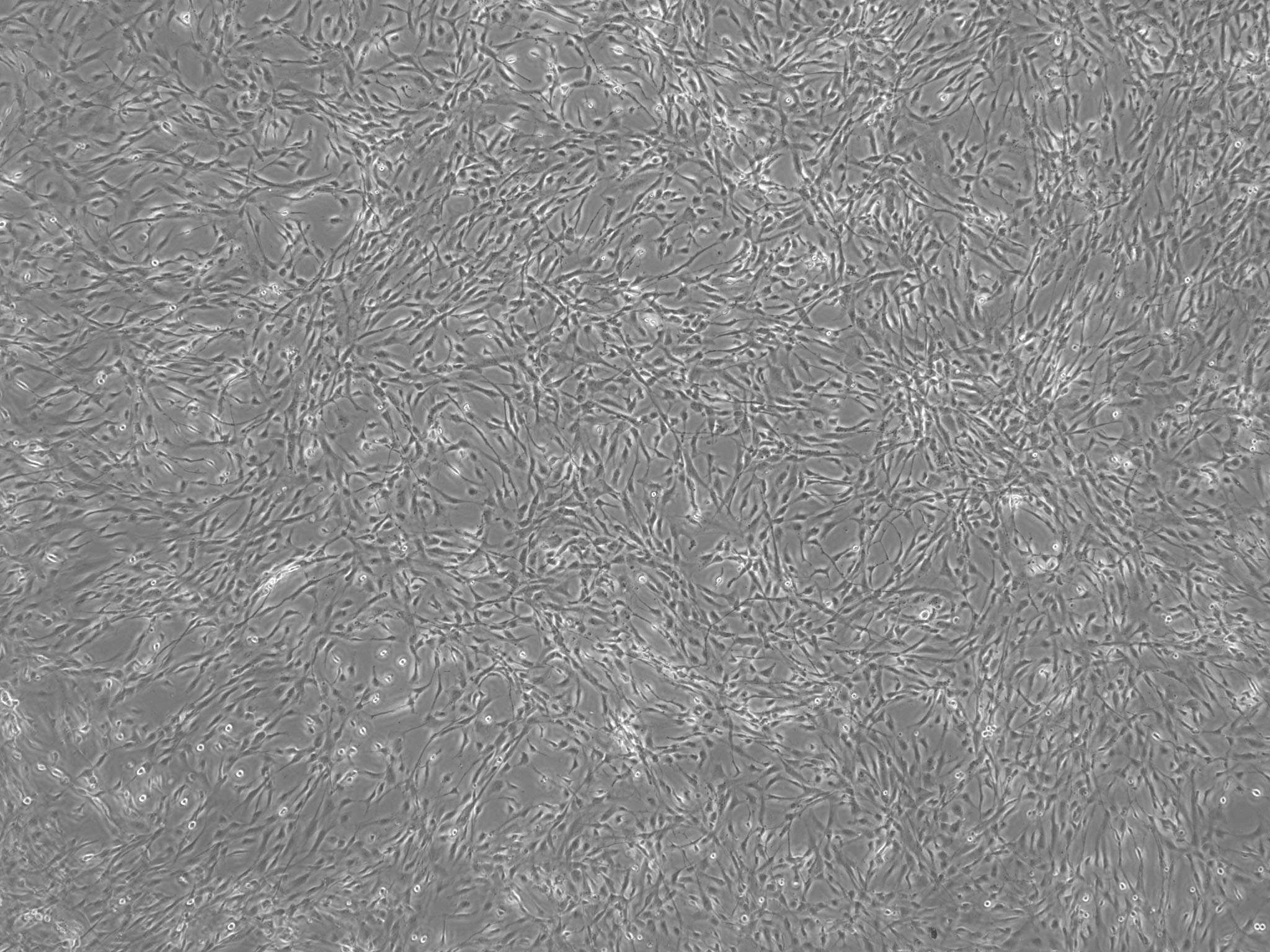

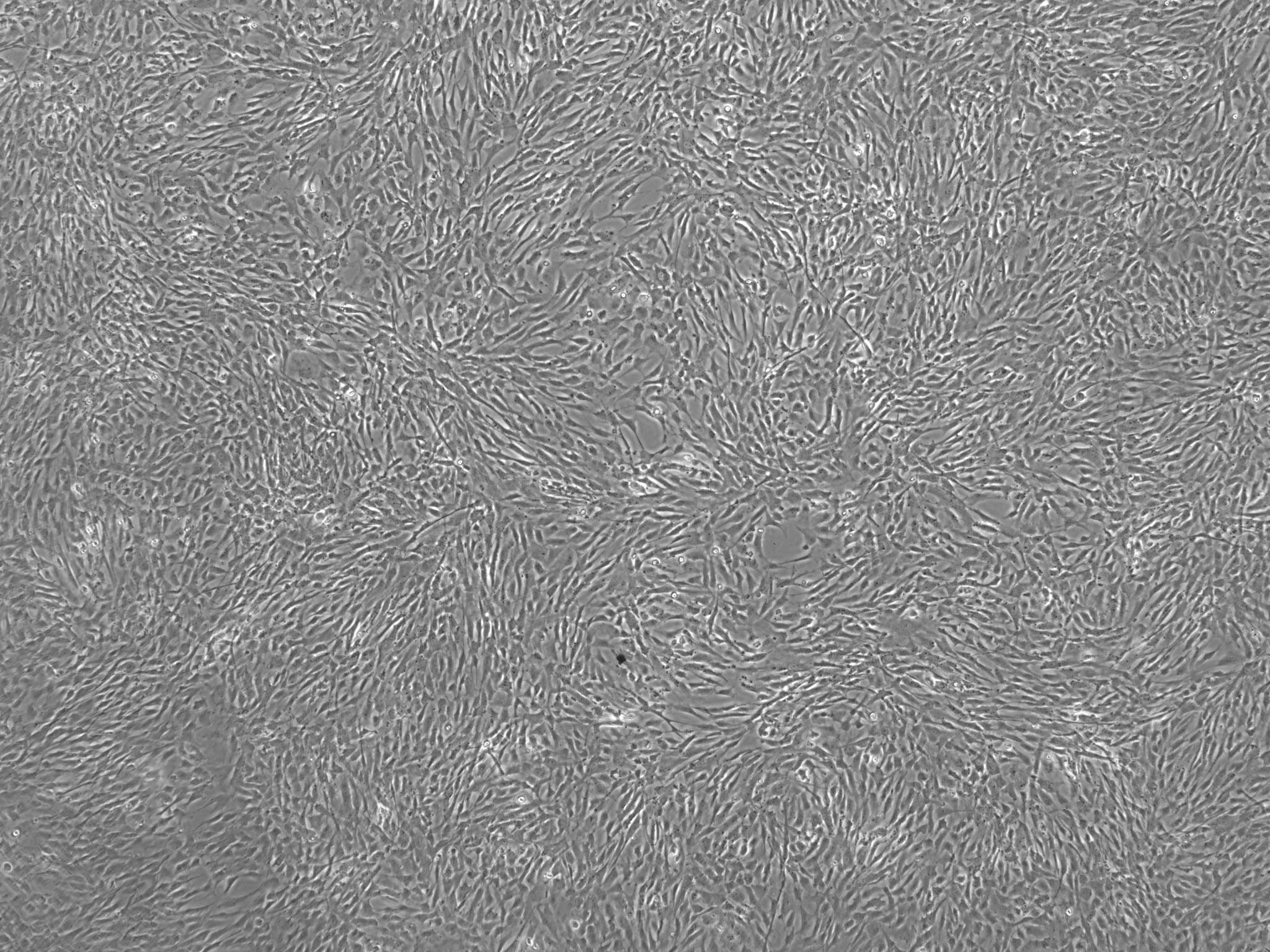

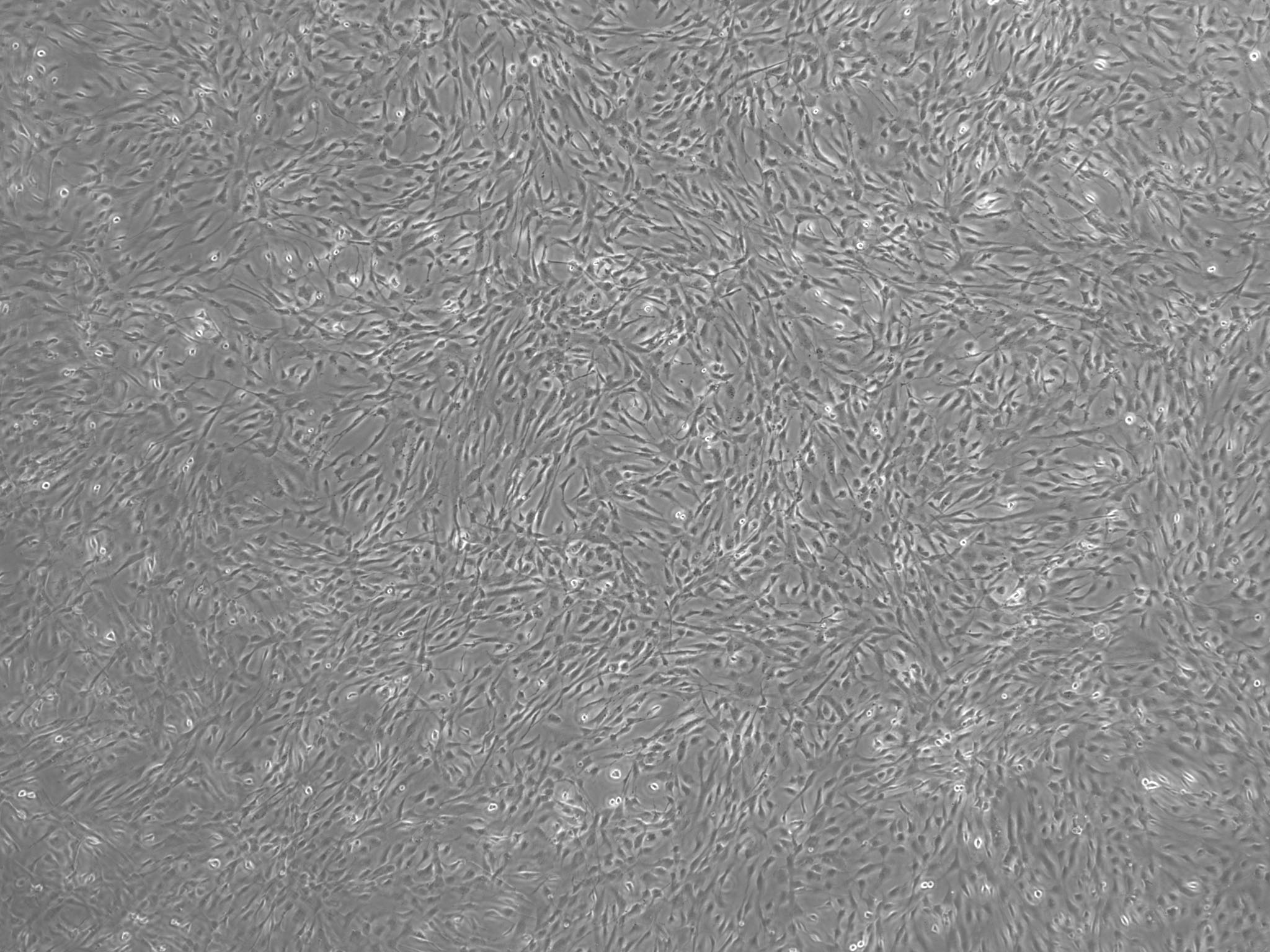


**Control T/I/C T/I/C + UB-SCG-51 (50 uM)**

**b. c.**

**
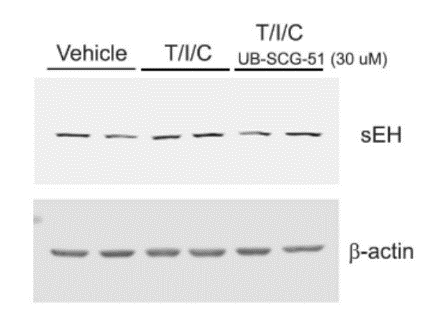
d. e.**

**
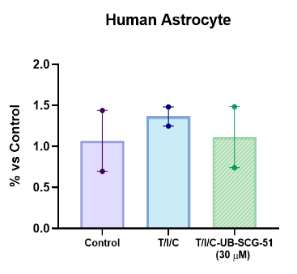
**

**
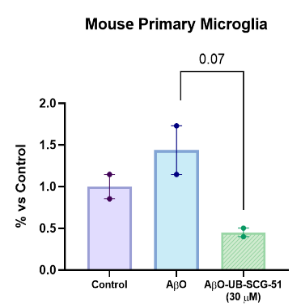
**

**
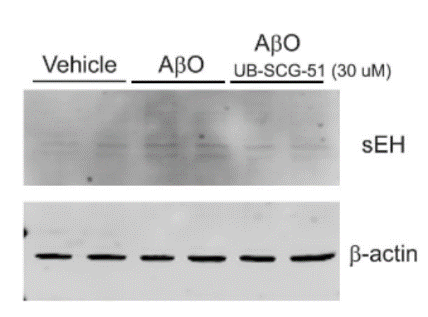
f. g.**

**Supplementary Table. S3 |** Pharmacokinetic parameters of UB-SCG-51.

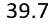


**Supplementary Table. S4 |** Parameters measured in PTZ test.

| **Compound** | **Mean time to clonic seizure latency in seconds (SEM)** | **Average clonic seizure (SEM)** | **Mean time to tonic seizure in seconds (SEM)** | **Protected from tonic/total (Mortality)** |
| --- | --- | --- | --- | --- |
| **Vehicle** | 253 (13.46) | 6.75 (0.75) | 851.50 (74.09) | 0/6 |
| **TPPU** | 605.33 (144.78) | 3.67 (0.56)** | 3063 (0.00)^****^ | 5/6^****^ |
| **UB-SCG-51** | 1006.5 (110.6)^**^ | 1.25 (0.25)^***,$^ | 0.00 (0.00)^****,$$$^ | 6/6^****^ |

Unpaired t-test or One Way ANOVA followed by Tukey post hoc analysis, vs Vehicle *p<0.05; **p<0.01; ***p<0.001; ****p<0.001; vs TPPU ^$^p<0.05; ^$$$^p<0.001).

**Supplementary Table. S5 |** KEGG pathway enrichment after UB-SCG-51 treatment.

**
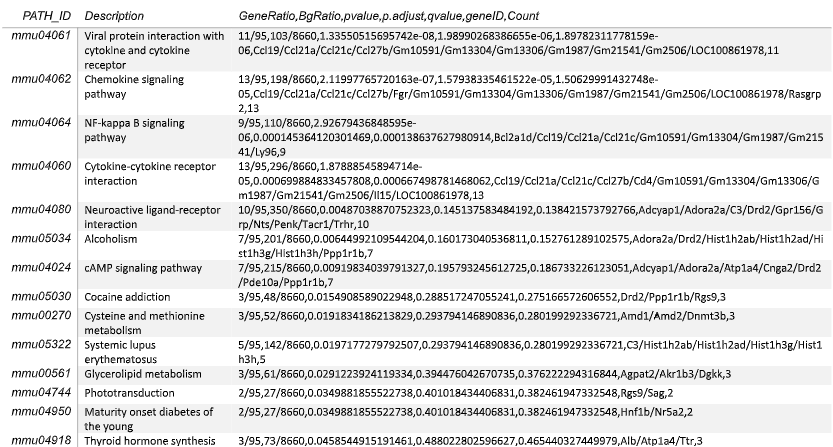
**
